# PDCD4 regulates axonal growth by translational repression of neurite growth-related genes and is modulated during nerve injury responses

**DOI:** 10.1261/rna.075424.120

**Published:** 2020-11

**Authors:** Andrés Di Paolo, Guillermo Eastman, Raquel Mesquita-Ribeiro, Joaquina Farias, Andrew Macklin, Thomas Kislinger, Nancy Colburn, David Munroe, José R. Sotelo Sosa, Federico Dajas-Bailador, José R. Sotelo-Silveira

**Affiliations:** 1 Departamento de Proteínas y Ácidos Nucleicos, Instituto de Investigaciones Biológicas Clemente Estable, Montevideo 11600, Uruguay; 2 Departamento de Genómica, Instituto de Investigaciones Biológicas Clemente Estable, Montevideo 11600, Uruguay; 3 School of Life Sciences, University of Nottingham, Nottingham NG7 2UH, United Kingdom; 4 Princess Margaret Cancer Centre, University Health Network, Toronto M5G 1L7, Canada; 5 University of Toronto, Department of Medical Biophysics, Toronto M5S 1A1, Canada; 6 Former Chief of Laboratory of Cancer Prevention at the National Cancer Institute-NIH at Frederick, Maryland 21702, USA; 7 Former Laboratory of Molecular Technologies, LEIDOS at Frederick National Laboratory for Cancer Research, Frederick, Maryland 21702, USA; 8 Departamento de Biología Celular y Molecular, Facultad de Ciencias UdelaR, Montevideo 11400, Uruguay

**Keywords:** programmed cell death 4 (PDCD4), axonal growth, axonal regeneration, translation, ribosome profiling

## Abstract

Programmed cell death 4 (PDCD4) protein is a tumor suppressor that inhibits translation through the mTOR-dependent initiation factor EIF4A, but its functional role and mRNA targets in neurons remain largely unknown. Our work identified that PDCD4 is highly expressed in axons and dendrites of CNS and PNS neurons. Using loss- and gain-of-function experiments in cortical and dorsal root ganglia primary neurons, we demonstrated the capacity of PDCD4 to negatively control axonal growth. To explore PDCD4 transcriptome and translatome targets, we used Ribo-seq and uncovered a list of potential targets with known functions as axon/neurite outgrowth regulators. In addition, we observed that PDCD4 can be locally synthesized in adult axons in vivo, and its levels decrease at the site of peripheral nerve injury and before nerve regeneration. Overall, our findings demonstrate that PDCD4 can act as a new regulator of axonal growth via the selective control of translation, providing a target mechanism for axon regeneration and neuronal plasticity processes in neurons.

## INTRODUCTION

The tumor suppressor programmed cell death 4 (PDCD4) protein was first described in cancer studies and has been shown to regulate protein synthesis by inhibition of EIF4A helicase activity (Yang et al. 2003; Suzuki et al. 2008; Matsuhashi et al. 2019) and via interaction with specific RNA motives present in a particular subset of target mRNAs (Loh et al. 2009; Wedeken et al. 2011; Biyanee et al. 2015). In mitogen stimulated cells, the degradation of PDCD4 is necessary for efficient protein translation, which is a prerequisite for cell growth and proliferation (Dorrello et al. 2006; Schmid et al. 2008). At present, while numerous molecules have been shown to regulate PDCD4, including *p21* (Göke et al. 2004), *Cdk4* (Jansen et al. 2005), and *JNK/c-Jun/AP-1* (Yang et al. 2003, 2006; Bitomsky et al. 2004), there is also a growing list of PDCD4 translational targets, including C-MYB, P53, SIN1, and BDNF (Singh et al. 2011; Wedeken et al. 2011; Wang et al. 2017; Li et al. 2020), together with internal ribosome entry site-regulated apoptosis inhibitors (Liwak et al. 2012). Although misregulation of PDCD4 in a variety of tumors (Zhang et al. 2006; Gao et al. 2007; Mudduluru et al. 2007; Zhen et al. 2016) suggests an important role in cancer development (Zhang et al. 2006; Gao et al. 2007; Mudduluru et al. 2007; Zhen et al. 2016), the full scope of PDCD4 translational targets and its potential role in other growth-dependent cellular systems has only recently started to be elucidated (Haas et al. 2020). In this regard, the molecular pathways involved in the development of tumor cells share a significant overlap with axonal growth and regeneration processes in the nervous system, particularly in the context of protein synthesis regulation (Chédotal et al. 2005; Heine et al. 2015).

The highly polarized nature that defines the morphology of a neuron makes local protein synthesis in the different cellular compartments (soma, dendrites and axons) an essential need for their development and function, being also important for plasticity and regenerative processes in the adult (Verma et al. 2005; Jiménez-Díaz et al. 2008; Huebner and Strittmatter 2009; Gumy et al. 2010; Park et al. 2010; Jung et al. 2011; Deglincerti and Jaffrey 2012; Kar et al. 2013; Obara and Hunt 2014; Sotelo-Silveira and Holt 2014; Ohtake et al. 2015; Terenzio et al. 2018). The acceptance of local protein translation as a key molecular mechanism in neuronal function has prompted the development of a variety of experimental models and omics approaches to investigate the specific axonal transcriptomes and proteomes (for review, see Farias et al. 2020). In this context, the elucidation of the regulatory pathways that can control the selective translation of axonal mRNAs has become an essential step in the understanding of neuronal development, growth and activity (Swanger and Bassell 2011; Jung et al. 2012). Among the various molecular mechanisms reported so far (Lin and Holt 2008; Holt et al. 2019), the mammalian target of rapamycin (mTOR) complex is described as a master regulator of local axonal translation and an important signaling process in axonal regeneration (Verma et al. 2005; Park et al. 2008; Terenzio et al. 2018), while also being affected in many different tumor types (Murray and Tee 2018). Interestingly, although PDCD4 protein has been described as an important downstream component of the mTOR pathway, it has not been directly associated with axonal processes. So far, the function of PDCD4 in the CNS has been linked to fetal alcohol syndrome, where it regulates general protein synthesis in cortical neurons (Narasimhan et al. 2013; Riar et al. 2014) and depression-like behaviors via BDNF regulation (Li et al. 2020). In spinal cord injury, PDCD4 has been shown to be down-regulated by microRNA-21 (Jiang et al. 2017) reinforcing the view of its potential role in neuronal mechanisms.

Considering the reported overlap in the molecular processes that promote both tumor and axon growth (Chédotal et al. 2005; Duman-Scheel 2009; Frank and Tsai 2009; Heine et al. 2015), we decided to investigate the potential role of PDCD4 in axonal function and regeneration. We hypothesized that as a repressor of translation, PDCD4 could be regulating mRNAs involved in axonal growth, regeneration and/or local protein synthesis and that its expression would be tightly regulated during these processes. Our study demonstrates how the manipulation of PDCD4 levels in central and peripheral nervous system neurons can control axonal growth, suggesting a potentially key role in axon regeneration in vivo. As a way to identify putative mRNAs regulated by PDCD4, we used ribosome profiling (Ribo-seq) to explore the translational effects of PDCD4 at a genome-wide level. We detected more than 250 possible mRNA candidates whose translational efficiency (TE) levels increase in the absence of PDCD4. Among them, we have identified a specific group of genes reported to be relevant in neurite/axonal development and regeneration. Overall, our findings demonstrate that PDCD4 can act as a new regulator of axonal growth via the selective control of translational targets, providing a specific mechanism for axon regeneration and neuronal plasticity processes in neurons.

## RESULTS

### Localization of PDCD4 in the nervous system

**FIGURE 1.**
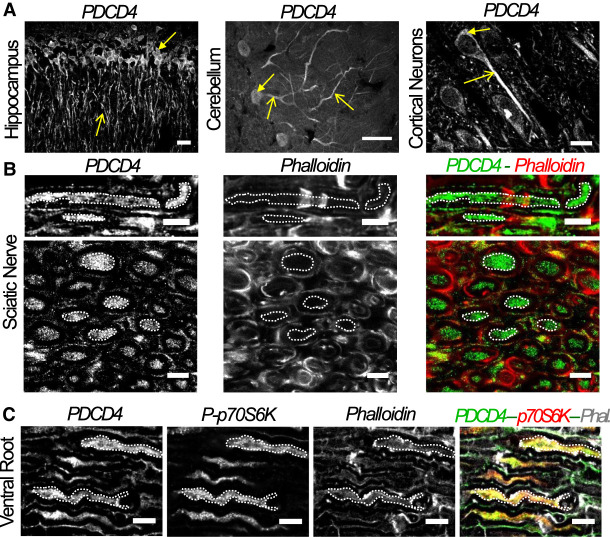
PDCD4 is expressed in dendrites and axons of the central and peripheral nervous system. (*A*) Immunohistochemistry assays show the distribution of PDCD4 protein at different types of neurons including CA1 hippocampal neurons, Purkinje of cerebellum and cortical neurons of prefrontal cortex of adult rats. Cell bodies are indicated by filled arrows and axons (or dendrite for Purkinje neurons) by unfilled arrows (scale bar, 20 µm). (*B*) PDCD4 protein is also detected in peripheral axons, like sciatic nerves, by immunohistochemistry. The images *above* correspond to longitudinal sections and the images *below* to transversal sections. Examples of axonal regions are highlighted in white dotted ROIs (scale bar, 10 µm). (*C*) Longitudinal sections of ventral roots. Examples of axonal regions are highlighted with white dotted ROIs. A partial colocalization between PDCD4 and p70-S6K signals is detected (scale bar, 5 µm).

To address the role of PDCD4 in neuronal cells, we first investigated its localization across different neuronal types. As shown in Figure 1A, we could detect PDCD4 protein in the central nervous system (CNS) of adult rats, both in cell bodies and neurites of CA1 hippocampal, Purkinje and cortical neurons. PDCD4 is also present in adult axons of the peripheral nervous system (PNS), as demonstrated following the analysis of its distribution in rat sciatic nerves, where specific axon detection can be more easily assessed. Crucially, this experimental approach allowed us to precisely detect high levels of PDCD4 inside the axoplasmic region of both longitudinal and transversal nerve cryosections (Fig. 1B).

Previous work in cancer cells demonstrated that both the translation and activity of PDCD4 can be regulated via the mTOR-p70S6K pathway (Dorrello et al. 2006), prompting us to investigate the potential link between PDCD4 and its upstream regulator p70S6K. As shown in Figure 1C, both PDCD4 and phosphorylated (active) p70S6K-Thr-389 are present in longitudinal sections of ventral root axons, evidence that the activated mTOR pathway colocalizes with PDCD4 in the axoplasm of peripheral neurons.

### Regulation of PDCD4 levels in primary neurons can control axonal growth

Following the demonstration of PDCD4 expression in CNS and PNS neurons, we decided to explore its functional role using neuronal in vitro models. First, we used primary cortical neurons isolated from embryonic mice. These cells can fully differentiate in culture to develop a morphologically intricate and functionally connected neuronal network after 10–12 d in vitro (Cotterill et al. 2016; Banker 2018). Following this period, axonal growth is decreased to allow the synaptic maturation that leads to the establishment of a functional network of connected neurons, which is observed after ∼2 wk in culture (Chiappalone et al. 2006).

**FIGURE 2.**
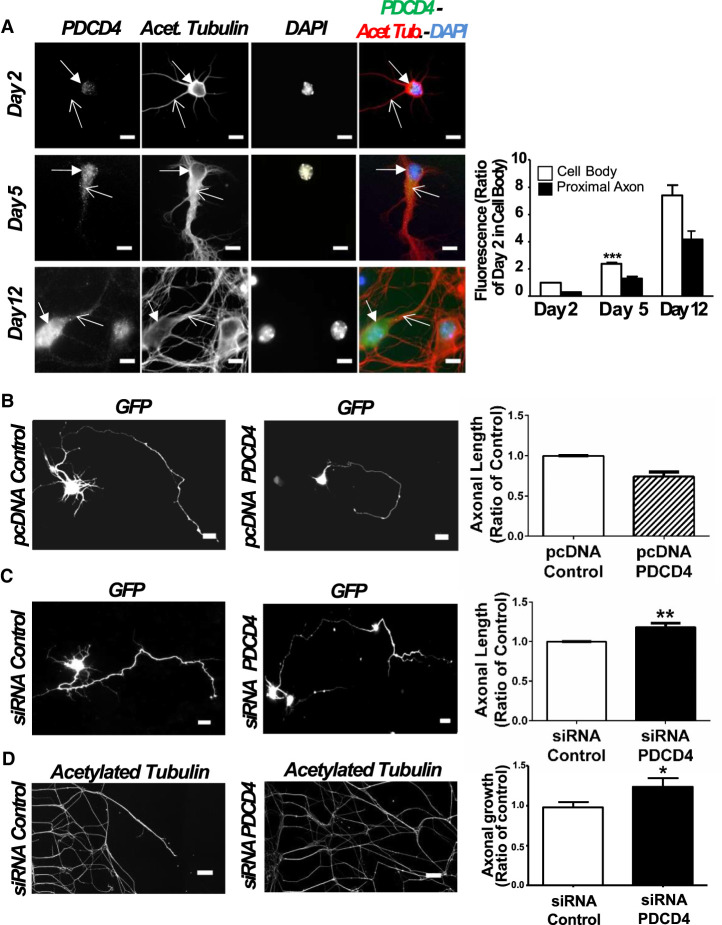
PDCD4 axonal levels change during neuron development and modulation of this protein can control axonal growth. (*A*) Immunocytochemistry assays show that PDCD4 levels increase during cortical primary neurons differentiation in vitro (scale bar, 20 µm). Cell bodies were indicated by filled arrows and axons by unfilled arrows. The signal quantification shows that cell bodies and axons have a significant increase in PDCD4 expression for day 5, and a trend to increase for day 12, always compared to day 2 ([***] *P* ≤ 0.001, ANOVA test with post-Tukey, error bars: SEM, *n* = 3 independent primary cortical neuron cultures for day 2 and day 5, with three technical replicates for each independent experiment; *n* = 2 for day 12, with two technical replicates for each independent experiment). (*B*) Cotransfected cortical primary neurons with a GFP plasmid and a PDCD4 plasmid, or a GFP plasmid and a pcDNA plasmid (scale bar, 20 µm). Overexpression of PDCD4 in transfected neurons at day 5 induce a decrease in axonal length (25%) compared to control condition ([**] *P* ≤ 0.01, paired test, *n* = 5 independent primary cortical neuron cultures, error bars: SEM). (*C*) Same as above but for PDCD4 knockdown using an siRNA for PDCD4 or an siRNA control (scale bar, 20 µm). Down-regulation of PDCD4 induces an increase in axonal length (18%) compared to the control condition ([**] *P* ≤ 0.01, paired *t*-test, *n* = 6 independent primary cortical neuron cultures, error bars: SEM). (*D*) Immunocytochemistry assays with acetylated tubulin in peripheral DRG neurons cultured in compartmentalized chambers and transfected with a permeable siRNA for PDCD4, or with a siRNA control (scale bar, 500 µm). Quantification of axonal growth shows similar effect as above: down-regulation of PDCD4 determines an increase (24%) of axonal growth increase (* *P* ≤ 0.05, Student's *t*-test, *n* = 4, error bars: SEM). In all cases, the “*n*” corresponds to independent biological replicates.

PDCD4 protein was detected in primary cortical neurons throughout their development in culture, with levels significantly increasing in cell body and axons between days 2 and day 5, and a major increase also detected after 12 d in vitro (Fig. 2A). The observation that PDCD4 levels increase in the late stages of neuronal network development and synaptic maturation (day 5–12) supports the hypothesis that increasing levels of PDCD4 could repress axon and/or neurite growth. To test this, we investigated the effect of PDCD4 overexpression on axonal growth following transfection with a PDCD4 plasmid at day 2 (24 h after seeding), with analysis of axonal length carried out 72 h later (day 5 of cell culture). Before the functional evaluation, we confirmed that neurons transfected with PDCD4 plasmid have a significant increase in PDCD4 levels detected by immunocytochemistry (Supplemental Fig. S1A). Increased PDCD4 levels during this period of active axonal growth (days 2–5 in vitro) produced a significant reduction in axonal length (Fig. 2B). To further confirm the dynamic regulation of axonal growth based on PDCD4 levels, we showed that the siRNA-dependent knockdown of PDCD4 produced the opposite effect, with a significant increase in axonal length (Fig. 2C). To assess if this effect was also observed in peripheral neurons, we evaluated axonal growth in dorsal root ganglia (DRG) neurons cultured in compartmentalized microfluidic chambers. Addition of a cell-permeable siRNA probe targeting PDCD4 produced a significant increase in axonal growth (Fig. 2D). Importantly, we confirmed that in both cortical and DRG neurons the addition of PDCD4 siRNA led to a significant decrease in PDCD4 levels detected by immunochemistry (Supplemental Fig. S1B,C), an effect further confirmed by immunoblotting using the neuroblastoma N2a cell line, which has high transfection efficiency (Supplemental Fig. S1D). Overall, these functional studies demonstrate that PDCD4 modulates axonal growth in central and peripheral nervous system neurons.

### Ribosome profiling reveals that PDCD4 regulates the translation of genes involved in axon/neurite growth

To explore the capacity of PDCD4 to regulate translation in neurons, we decided to use the ribosome profiling strategy (Ribo-seq) in differentiated PC12 neuron-like cells as a suitable and relevant model for the investigation of neuronal mechanisms (Shao et al. 2016; Zheng et al. 2016). To confirm the experimental validity of this approach, we first demonstrated the expression of PDCD4 in PC12 cells at different time points following NGF-induced neuron differentiation, with levels remaining relatively stable throughout the culture period (Supplemental Fig. S2A). Using a doxycycline inducible shRNA system, we obtained stable cell populations with inducible silencing of PDCD4 expression. In this way, addition of NGF and doxycycline for 72 h allowed us to achieve neuron differentiation of PC12 and silencing of PDCD4 expression (Supplemental Fig. S2B–G).

**FIGURE 3.**
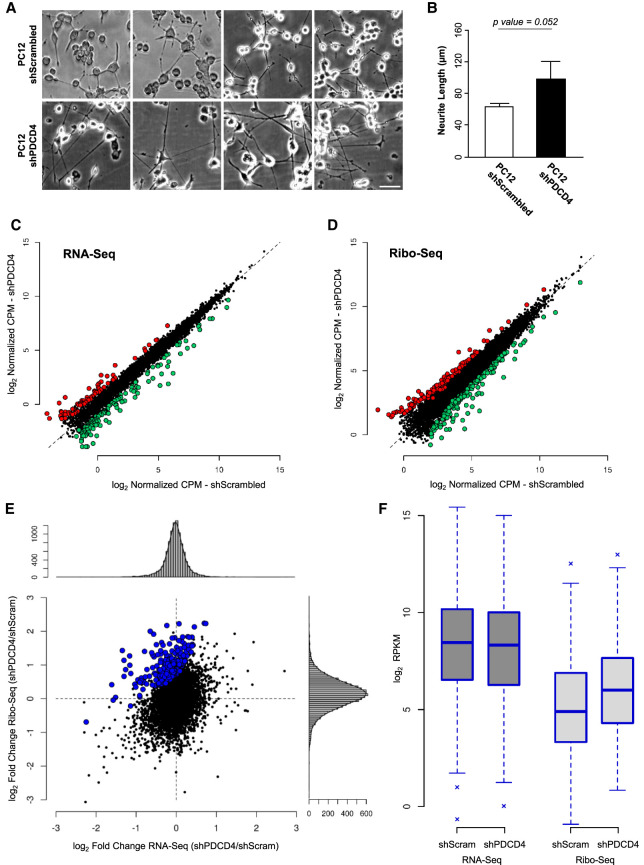
Translatome and steady-state transcriptome levels of expression for mRNAs was quantified genome-wide by Ribo-seq and RNA-seq, respectively, in absence (shPDCD4) and presence (shScrambled) of PDCD4 achieved by lentiviral transfection in neuron-like differentiated PC12 cells. (*A*) To explore if PDCD4 modulates neurite outgrowth also in differentiated PC12 cells, we compared neurite length in differentiated PC12 growing in presence and absence of PDCD4. Three independent cultures were contrasted and illustrative fields are shown (scale bar, 50 µm). (*B*) Quantification of neurite length of *A* is shown and an increase of almost 1.6× in absence of PDCD4 is detected, with a marginal trend toward significance (*P* = 0.052, Student's *t*-test, *n* = 3 independent cell cultures with an average of 80 neurites considered by replicate, error bars: SD). (*C*) Scatter plot showing PDCD4 regulation at the level of transcriptome evaluated by RNA-seq. Red and green dots indicate differentially expressed genes, up- and down-regulated, respectively (|fold change| > 2 and *P* < 0.05 estimated by *edgeR*). (*D*) Same as *C* but for PDCD4 regulation at the level of translatome evaluated by Ribo-seq. (*E*) Fold changes (shPDCD4/shScrambled) at transcriptome and translatome levels are contrasted for detected genes. Possible PDCD4 translational targets are those that present a significant increase in translational efficiency (*P* < 0.05 estimated by *Xtail*), and are indicated in blue (267 mRNAs). Along the scatter plot, *vertical* and *horizontal* histograms show fold change values distribution estimated by Ribo-seq and RNA-seq. (*F*) RPKM gene expression levels for PDCD4 translational targets indicated in blue in *E* are shown for the two compartments (RNA-seq and Ribo-seq) in each condition.

As a first experimental approach to the use of PC12 neuron-like cells, we analyzed if neurite growth was regulated by the presence or absence of PDCD4. Confirming our previous observations in primary neuron cultures, knockdown of PDCD4 in PC12 cells increased neurite length compared to controls (Fig. 3A,B), allowing us to validate their use in the search for putative PDCD4 translational targets. For this, we isolated polysomal and total RNA from differentiated PC12 cells in the presence and absence of PDCD4. Samples were analyzed using parallel RNA-seq and Ribo-seq protocols (Supplemental Fig. S3A–C), which allowed us to determine steady-state transcriptome and translatome levels for over 10,000 mRNAs (Supplemental Table S1). Fold changes (shPDCD4/shScrambled) at both transcriptome and translatome levels were contrasted for detected genes, and according to its previously described role (Matsuhashi et al. 2019), PDCD4-knockdown has an impact on both transcriptome and translatome compartments (Table 1; Fig. 3C,D; Supplemental Fig. S3D–G). To specifically study PDCD4 regulation in protein synthesis, we compared translation efficiency (TE) levels between conditions. TE, which is calculated as the ratio between translatome over transcriptome levels for a particular mRNA, is an informative parameter to discriminate translational regulation events from those exclusively transcriptional, and indicates how efficiently an mRNA is translated (Ingolia et al. 2009). Using the *Xtail* R package (Xiao et al. 2016) to explore TE differences, we detected 267 mRNAs whose TE levels significantly increase following PDCD4 knockdown (*P* < 0.05; Table 1; Fig. 3E,F; Supplemental Fig. S4; Supplemental Table S2), and 100 mRNAs whose TE decreases. The first group, which represents potential PDCD4 targets, displays unaltered steady-state transcriptome levels (88% has |fold change| <1.5-fold), but they increase their levels of translation (86% has fold change >1.5-fold; Fig. 3F). In order to explore if the regulation of translational efficiency was also being observed at the protein level, total protein samples obtained at the same time of the sequencing analysis were quantified by label-free proteomics. Although we observed some specific correlations with Ribo-seq data, we could only detect a small fraction of proteins changing significantly, with the sensitivity of this approach not sufficient to detect global correlations (Supplemental Fig. S5).

**TABLE 1.**
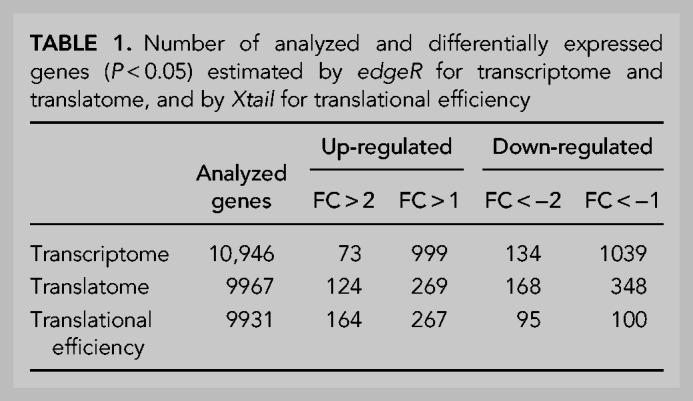
Number of analyzed and differentially expressed genes (*P* < 0.05) estimated by *edgeR* for transcriptome and translatome, and by *Xtail* for translational efficiency

To investigate the functional implications of PDCD4 regulation over the 267 putative targets that increase their translational efficiency, protein association analysis was performed using STRING (Jensen et al. 2009). This revealed three to four related and altered protein cores and a network with significantly more interactions than expected (*P*-value = 0.0163; Supplemental Fig. S6A). The observed related protein cores within PDCD4 putative targets are grouped under *regulation of mitosis* (Moustafa-Kamal et al. 2019), *centromere and kinetochore*; *regulation of transcription and splicing* (Kim et al. 2014; see Fig. 3C; Supplemental Fig. S3D,E); *regulation of mitochondrial activity* (Zhang et al. 2006); and *protein translocation to the endoplasmic reticulum* (Hudson 2008; Wang et al. 2013). Interestingly, when analyzing the list of targets whose translational efficiency decreases in the absence of PDCD4 (100 genes), we found that neither core-related proteins nor protein interactions were significantly altered (*P*-value = 0.954; Supplemental Fig. S6B). This difference provides a good indication that those putative targets increasing their translation after PDCD4 knockdown represent a defined set of cellular functions, while those down-regulated in our sequencing data are likely emerging as a secondary cell effect and/or experimental noise.

**FIGURE 4.**
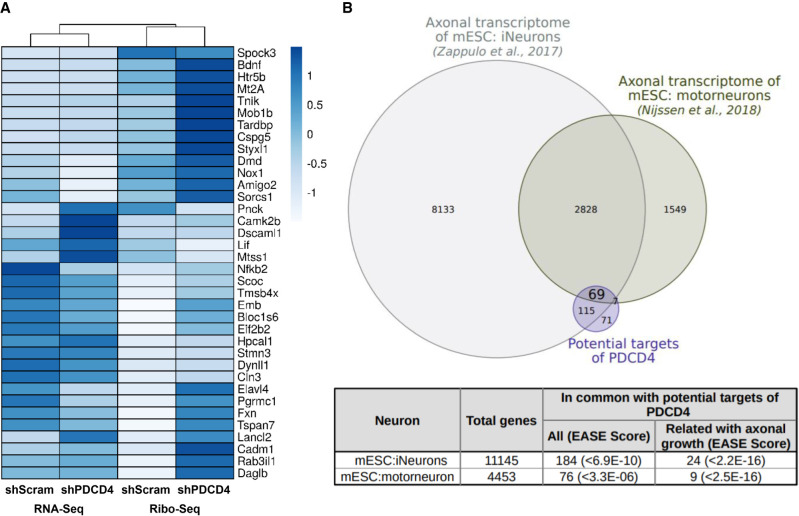
PDCD4 regulates translation of several neurite growth-related genes evidenced by Ribo-seq and RNA-seq. The putative PDCD4 targets are present in previously described axonal transcriptomes. (*A*) Expression of neurite and axon growth-related genes is represented by a heatmap, where an induced expression at translation level is detected in the absence of PDCD4. (*B*) Venn diagram showing the intersection between the potential PDCD4 targets and axonal transcriptomes derived from mESc (Zappulo et al. 2017; Nijssen et al. 2018). Separate and overlapping expressions between samples are shown. Only transcripts with a level of expression of TPM ≥ 1 were considered. The table shows the type of neuron used in each study, the total genes detected and the number of common genes between potential PDCD4 targets (or potential PDCD4 targets related to axonal growth) and each axonal transcriptome. The EASE Score (a modified Fisher exact *P*-value) is also shown, which indicates gene enrichment.

In order to uncover those potential PDCD4 targets with functional links to neurite, axon and/or dendritic growth, we curated the list using in-house software that allowed us to link published articles with gene lists and user-defined terms (Radío S, Sotelo-Silviera JR, and Smircich P, in prep.; https://github.com/sradiouy/IdMiner). We also explored the list of differentially expressed genes separately at the transcriptome or translatome level (fold change >2 and *P*-value <0.05 estimated by *edgeR*), and the down-regulated genes at TE (100 genes with *P*-value <0.05 estimated by *Xtail*), searching for genes that might inhibit neurite outgrowth. This comprehensive approach highlighted several experimentally validated axon and/or neurite outgrowth-related genes from the list of potential PDCD4 translational targets. This signature of 36 genes was mainly composed of up-regulated genes at the level of TE in PDCD4 absence (26 genes), but also by genes up-regulated only at the translatome (four genes) or transcriptome levels (three genes), or down-regulated at TE (three genes) whose ascribed function is to inhibit neurite outgrowth. Transcriptome and translatome levels of the 36 genes show a global induction of translation after PDCD4 knockdown, from low-middle to high translational levels (Fig. 4A).

Among the signature of neurite and axon-growth-related genes, we found genes previously reported to directly control neurite outgrowth: *Elavl4*, *Styxl1*, *Bdnf*, *Dmd, Lancl2, Lif*, and *Nfkb2*. In the case of ELAVL4 (also known as HUD), DMD and LANCL1 proteins, their expression is required for neurite outgrowth in PC12 cells (Mobarak et al. 2000; Acosta et al. 2004; Fukao et al. 2009; Zhang et al. 2009), while the pseudophosphatase MK-STYX (STYXL1) increases both the number of cells with neurite extensions and neurite outgrowth in PC12 (Flowers et al. 2014). Brain-derived neurotrophic factor (BDNF) was shown to increase neurite length in rodent primary neuronal cultures (Iwasaki et al. 1998; Cohen-Cory et al. 2010; Dajas-Bailador et al. 2012), as well as in PC12 cells (Squinto et al. 1991; Iwasaki et al. 1997). On the other hand, the TE of LIF is down-regulated in PDCD4's absence, and it was shown that activating LIF receptor signaling can have a negative impact on neurite extension in PC12 cells (Ng et al. 2003). Particularly interesting is the relationship between the transcription factor complex nuclear factor-kappa-B (NFκB) and neuritogenesis, where activating the NFκB pathway and increasing *Nfkb2* gene expression promotes neuritogenesis in PC12 cells (Manecka et al. 2013). This strong link motivated us to confirm PDCD4 regulation of NFKB2 protein abundance. For this we used a model of cortical neurons in culture, silencing PDCD4 by a new set of lentiviral particles with shRNA against PDCD4, and quantifying protein abundance by western blot. By this, we confirmed by an orthogonal method that NFKB2 total protein levels increase significantly after knockdown of PDCD4 (Supplemental Fig. S7).

In line with the notion that PDCD4 can regulate axon-related mRNA translation in neurons, we found that a significant proportion of the 267 putative translational targets identified in our Ribo-seq analysis are found in two different published axonal transcriptomes from in vitro neuron models (Fig. 4B). On the other hand, those genes for which we observe a decrease in the TE after PDCD4 knockdown are not specifically enriched in axonal transcriptomes, providing further support to our hypothesis that they reflect secondary mechanisms (Supplemental Fig. S8). A significant fraction of PDCD4 mRNA translational targets were also found in the recently described in vivo motor axon transcriptome (Farias et al. 2020) (23 out of 255, in a set of 1008 mRNAs; *P*-value = 2.8 × 10^−04^). These findings support the idea that PDCD4 could also be a player in regulating local axonal responses and plastic processes in vivo.

### Sciatic nerve injury in vivo reduces local axonal levels of PDCD4

**FIGURE 5.**
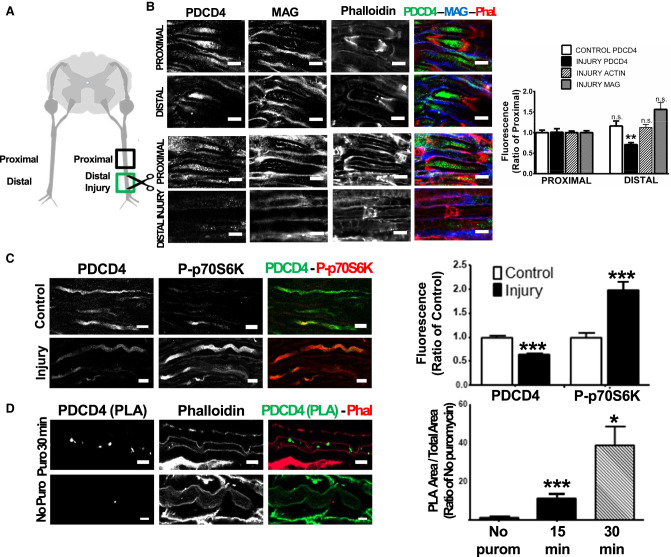
Sciatic nerve injury induces a decrease of PDCD4 levels locally in axons next to the injury site and an up-regulation (and activation) of p-p70S6K, a component of mTOR-PDCD4 pathway. PDCD4 could be locally synthetized in peripheral axons as a possible mechanism for protein level regulation. (*A*) A complete transection of the sciatic nerve was performed in adult rats using the contralateral nerve as a control condition. The analyzed regions are labeled as proximal and distal (or distal injury) in reference to cell bodies. (*B*) Eighteen hours post injury, the sciatic nerves were extracted from the animals and the levels of PDCD4 were analyzed by immunohistochemistry (scale bar, 5 µm). Signal quantification shows no differences on PDCD4 levels between regions in the control condition (distal vs. proximal), but in the injury to the sciatic nerves, a significant decrease in PDCD4 levels at the injury region was detected (distal injury vs. proximal). These changes are specific to PDCD4 because other proteins in the axon-like F-Actin (evidenced by phalloidin) and MAG protein did not have differences on expression levels comparing distal injury versus proximal (**P ≤ 0.01, two-tailed Mann–Whitney test, *n* = 3, error bars: SEM). White dotted ROIs correspond to examples of quantified axonal regions. (*C*) As in *B*, levels of PDCD4 and p70S6K were analyzed by immunohistochemistry comparing injury axons vs control (scale bar, 10 µm). By signal quantification we detect an increase in the phosphorylated form of p70S6K, a direct upstream regulator of PDCD4 expression on the mTOR pathway (***P ≤ 0.001, two-tailed Mann–Whitney test, *n* = 2, error bars: SEM). White dotted ROIs correspond to examples of axonal quantified regions. (*D*) Maximum intensity projection images showing Puro-PLA signal for PDCD4 inside the axoplasm at 30 min of puromycin incubation and the associated control condition without puromycin. The green spots inside the axoplasm reveals that PDCD4 is newly synthetized in the axon. The *right* panel shows the signal quantification at two different time points of puromycin incubation compared to the control (***P ≤ 0.001 and *P ≤ 0.02, ANOVA and Mann–Whitney test, *n* = 2, error bars: SEM). White dotted regions define the limits of the axoplasm region. Note that in all the cases, “*n*” are from independent biological replicates.

Our in vitro functional studies and ribosome profiling data support the hypothesis that PDCD4 is a novel regulator of axonal function. For this reason, we decided to test our findings in vivo, using a model of axon regeneration, a cellular process where the regulation of axonal protein synthesis is important (Verma et al. 2005; Huebner and Strittmatter 2009; Gumy et al. 2010). For this, we performed a full transection of the rat sciatic nerve, using the contralateral nerve as a control for no injury (Fig. 5A). At 18 h post injury, when the nerve is in the active regeneration phase, we analyzed the levels of PDCD4 along the axoplasm. Our analysis demonstrates that uninjured controls show no change in the expression of PDCD4 along the axoplasm of the sciatic nerves (Fig. 5B, confocal images of “Proximal” and “Distal” axonal sections in relation to the cell bodies). However, we observed a significant decrease of ∼30% of PDCD4 axonal levels at the site of injury (“distal injury” in diagram) when compared to the proximal region of the same nerve segment (Fig. 5B). Importantly, these changes in PDCD4 levels are not due to an overall loss of protein content at the site of injury, as the levels of actin and MAG do not change when comparing all conditions (Fig. 5B). These findings allowed us to conclude that axonal levels of PDCD4 are dynamically decreased at the site of axonal injury and during the regeneration process.

The potential role of PDCD4 as a translation regulator in axonal growth would require the existence of precise molecular pathways controlling its localized expression. In this regard, degradation of PDCD4 would be able to rapidly promote axon regeneration in the context of nerve injury. Indeed, concomitant to the decrease in PDCD4 levels in injured axons, we found a significant increase in the activated form of p70S6K (phospho-p706SK), which acts as a direct regulator of PDCD4 degradation via the mTOR pathway (Fig. 5C; Dorrello et al. 2006). The tight control of protein levels in the axoplasm is not only dependent on degradation, but also on localized protein translation (Vuppalanchi et al. 2009; Yoon et al. 2009; Sahoo et al. 2018). Indeed, PDCD4 mRNA levels are detected in the axon of peripheral neurons, both in vivo (Farias et al. 2020) and in vitro in our DRG compartmentalized cultures, where qPCR analysis of RNA extracted from the axon compartment showed an increase from day 3 to 12 (day 3 *C*_t_ value = 25.5 and day 12 *C*_t_ value = 23.6) samples. To test if local translation could control PDCD4 levels in axons, we carried out the Puromycin-Proximity Ligation Assay or Puro-PLA in rat ventral roots in vivo (tom Dieck et al. 2015) to explore local protein synthesis. Consistent with its dynamic role in the control of axon processes, our results indicate that PDCD4 can be locally translated in mature noninjured axons (Fig. 5D). Overall, these results revealed two possible molecular mechanisms for the regulation of PDCD4 levels in mature axons ex vivo, either via degradation and/or local protein synthesis.

## DISCUSSION

Our studies demonstrate that PDCD4 is expressed in different neuronal cell types of the CNS and PNS and is distributed in axonal and dendritic compartments, possibly interacting with other components of the mTOR pathway, such as its upstream protein regulator p70S6K (Fig. 1). Since PDCD4 is known to regulate protein synthesis in tumor models and also act as a downstream component of the mTOR pathway, we postulated that it could have a role in molecular processes relevant to axonal function. The possibility that PDCD4 functions as a translation regulator factor in both cancer and neuronal cells agrees with the observation that many of the key cellular hallmarks of cancer encompass molecular processes that are crucial in nervous system development, such as invasive cell growth, cytoskeleton rearrangements, ECM dynamic interactions and survival (Duman-Scheel 2009; Heine et al. 2015).

In mouse primary cortical neurons, we observed that PDCD4 is localized to the cell body and axons, with levels increasing during time in culture (Fig. 2A). This increase in PDCD4 levels correlates with neuronal network maturation and the establishment of synaptic contacts, a period that requires decreased axon growth. It is thus possible that elevated levels of PDCD4 mark the end of active axonal growth and the establishment of a stable cortical network. Further confirmation came from the overexpression of PDCD4, which caused a significant decrease in axonal growth. Importantly, the knockdown of PDCD4 levels caused the opposite effect, with a significant increase in axonal length (Fig. 2C,D). The analysis of axonal growth in primary cortical neurons constituted a useful approach for the investigation of the cellular and molecular mechanisms that control neuronal network development in the CNS. However, the elucidation of those mechanisms that can control axonal growth is also a fundamental area of research in the study of nerve regeneration in PNS (He and Jin 2016). It was thus important to investigate if PDCD4 could have a role in the regulation of axonal growth using an in vitro model of peripheral neurons, such as dissociated DRG primary neurons. Consistent with the high levels of PDCD4 observed in peripheral axons in vivo (Fig. 1B,C), and the functional data from cortical neurons (Fig. 2B,C), siRNA-dependent knockdown of PDCD4 in DRG neurons grown in microfluidic chambers (Taylor and Jeon 2011; Dajas-Bailador et al. 2012) showed a significant increase in axon growth (Fig. 2D). Overall, the functional studies in primary neurons of central and peripheral origin demonstrate a clear mechanistic link between the control of PDCD4 levels and the regulation of axon growth and network development, confirming that PDCD4 can modulate these processes, probably at the level of translation.

In order to explore the molecular signature underlying the link between PDCD4 and the control of neurite/axon growth, we decided to use a genomic approach to elucidate putative PDCD4 translational targets. To do this, we first generated a stable PC12 cell line able to induce PDCD4 knockdown by shRNA (Supplemental Fig. S2), and then confirmed that similar to primary neurons, PDCD4 knockdown caused an increase in the neurite length of differentiated PC12 neuron-like cells (Fig. 3A,B). Despite its described role in selective mRNA translational mechanisms, the investigation of PDCD4 targets has been largely unexplored using genomics approaches, neither in cancer nor in neuronal models. However, very recent work by Haas and collaborators described PDCD4 targets using transcriptomics and Ribo-seq in a telomerase-immortalized human epithelial cell line (Haas et al. 2020). The implementation of Ribo-seq protocols, which are based on deep sequencing of ribosome-protected mRNA fragments, makes it possible to monitor translation directly, significantly improving the estimation of protein translation levels as compared to the classic RNA-seq approaches (Ingolia et al. 2012; Eastman et al. 2018). Our Ribo-seq data confirmed the key role of PDCD4 in the regulation of translation and provided only the second approximation to the full scope of its cellular targets and the first in a neuronal cell model (Fig. 3C–F). It allowed us to compare the PDCD4-dependent transcriptome and translatome data sets (Fig. 3C,D), identifying 267 mRNAs with a potential role as PDCD4 translational targets (Fig. 3E,F). This set of neuronal-related putative targets significantly increases their translational efficiency in the absence of PDCD4 (*P*-value <0.05 estimated by *Xtail*; Supplemental Table S2). We compared this list with that defined by Haas et al., where 62 mRNAs increase their translation upon PDCD4 silencing. Although 50 of those 62 targets could be detected in our study and show considerable expression levels, only *Cldnd1*, an apoptosis-related gene in epithelial cells (Achari et al. 2015), is common to both studies. This is likely the reflection of differences in cell type, developmental stage and experimental model, and provides a tantalizing perspective on the regulatory potential that can be attributed to PDCD4 and which is likely controlled by the additional recruitment of cell- and stage-specific mechanisms.

In order to further elucidate the regulatory mechanisms at play, samples from the same RNA-seq and Ribo-seq experimental protocols were analyzed by label-free quantitative proteomics (LFQ). However, the number of observed and regulated proteins (∼3000 and 141 proteins, respectively) were lower than expected. From 141 differentially expressed proteins (*P*-value <0.05; ANOVA), 87 of them (62%) show the same direction of change seen in the Ribo-seq data (with similarly positive or negative fold changes; Supplemental Fig. S5B). In addition to the lack of sensitivity, it is also likely that protein levels might need to be evaluated at later time points than the Ribo-seq data, but this was beyond the scope of this study. Ultimately, protein levels are also subject to dynamic regulatory processes that control their half-life and degradation, and which might prevent the direct correlation of Ribo-seq data with estimation of proteome levels, particularly for low expression genes, which could be relevant in PDCD4 function (Zubarev 2013; Kumar et al. 2016).

Based on our functional results with primary neurons, we investigated the list of potential PDCD4 targets identified from our Ribo-seq data to uncover a neurite/axon growth gene signature. A list of 36 PDCD4 targets emerged with axon growth and/or neurite outgrowth links (Fig. 4A). Besides the genes associated with neurite outgrowth in PC12 cells described previously, the remaining genes were also described as associated with neurite or axonal growth previously in the literature (Bisogno et al. 2003; Nakanishi et al. 2006; Su et al. 2006; Ghiani et al. 2010; Fiesel et al. 2011; Bali et al. 2013; Kar et al. 2013; Olbrich et al. 2013; Mincheva-Tasheva et al. 2014; Munnamalai et al. 2014; Siddiq et al. 2015; Yu et al. 2016; Parviainen et al. 2017; Tang et al. 2017; Chen et al. 2019). Relevant and interesting examples include genes coding for proteins associated with the cytoskeleton, either actin with *Tmsb4x* (van Kesteren et al. 2006; Yang et al. 2008) or microtubules with *Stmn3* (Riederer et al. 1997; Manna et al. 2007); cell adhesion molecules like *Cadm1* (Nagara et al. 2012), *Amigo2* (Kuja-Panula et al. 2003), *Dscaml1* (Hattori et al. 2008; Zhu et al. 2013; Hutchinson et al. 2014), and *Emb* (Lain et al. 2009), transmembrane proteins associated to signal transduction including *Tspan7* (Bassani and Passafaro 2012); calcium-binding proteins like *Spock3* (Schnepp et al. 2005; Yamamoto et al. 2014), *Hpcal1* (Braunewell et al. 2011; Wang et al. 2014), and *Camk2b* (Fink et al. 2003; Yan et al. 2016), anterograde and retrograde transport and signaling with mRNAs like *Dynll1* (Lin et al. 2015) and *Lancl2*, different kinases like *Tnik* (Kawabe et al. 2010), *Mob1b* (Lin et al. 2011; Song et al. 2018), *Pnck* (Wayman et al. 2004; Uboha et al. 2007), and also synaptic-associated proteins like *Rab3il1* (Villarroel-Campos et al. 2016). Importantly, a significant proportion of PDCD4 putative translational targets identified in our analysis were also found in axonal transcriptomes from in vitro neuron models (Zappulo et al. 2017; Nijssen et al. 2018) and in vivo motor axon transcriptome (Farias et al. 2020) (Fig. 4B), supporting the validity of these processes beyond our experimental models.

Indeed, our own work managed to extend the observation of PDCD4's capacity for regulation of axon growth in primary neurons to an in vivo axon regeneration model, demonstrating that PDCD4 levels decrease following injury and during the regenerative growth phase (Fig. 5A,B). Taking into consideration that PDCD4 levels are high along adult peripheral axons in sciatic nerves (Fig. 1B), we believe a decrease in the levels of this translational repressor would allow the expression of growth-related proteins to aid local axon regeneration. Taken together, our in vitro and in vivo experiments support the idea that the dynamic control of PDCD4 levels in the neurons and axons could act as a new regulatory mechanism of protein synthesis in a specific and growth-oriented manner.

The role proposed for PDCD4 in the regulation of local protein synthesis in neurons would require a tight control of its levels in the axon. In agreement with previous reports (Verma et al. 2005; Terenzio et al. 2018), we found that the active form of p70S6K protein (phosphorylated at Thr 389) was increased twofold at the injury site, when compared to uninjured axons (Fig. 5C). Given that the local activation of the mTOR pathway in the injured axons increases axonal protein synthesis (Verma et al. 2005; Terenzio et al. 2018), our findings confirm that the increased phosphorylation of p706SK in the axons correlates with a decrease in PDCD4 levels, likely mediated by activation of the proteasome system (Dorrello et al. 2006). In this scenario, the translation repression offered by PDCD4 would be removed, releasing the potential for growth mechanisms.

Local depletion of PDCD4 would be useful to mediate a rapid regeneration response; however, local neosynthesis can be used as an important mechanism to modulate protein synthesis of specific targets in other contexts of neuron development, when axon growth is reduced and synaptic consolidation might be needed. Indeed, PDCD4 levels have been shown to be regulated by miR-21 in cancer models increasing transformation, invasion, and metastasis (Asangani et al. 2008; Matsuhashi et al. 2019). Consistent with this, miR-21 is up-regulated during axonal growth and regeneration in DRG axons (Strickland et al. 2011) and miR-21 and PDCD4 have been analyzed in a model of spinal cord injury, where whole tissue levels of miR-21 increase and PDCD4 decrease after injury (Jiang et al. 2017). To test the potential existence of local protein synthesis as a regulatory mechanism controlling PDCD4 protein levels in the axoplasm, we used proximity ligation assays (PLA) (tom Dieck et al. 2015). Our results suggest that PDCD4 is locally translated in the axoplasm of peripheral neurons (Fig. 5D), supporting previous neurite data of newly synthetized proteomics, where PDCD4 is also detected (Zappulo et al. 2017).

Overall, our study shows the expression of PDCD4 in different types of neurons ex vivo, in vitro and also at different development stages in both mouse and rat experimental models. These results demonstrate a role of PDCD4 in processes where the dynamic regulation of protein synthesis is of crucial importance, such as axonal growth, in both development and regeneration. We report the first Ribo-seq data set for PDCD4 in a neuronal model, defining 267 mRNAs that could be regulated by PDCD4 at the translational level, with a significant number of these being related to neuronal plasticity and axonal growth processes. The potential relevance of this regulatory capacity for PDCD4 is further suggested by its regulation by local protein synthesis and/or degradation in peripheral axons. Taken together, our findings uncover a new role for PDCD4 in protein synthesis regulation at neuronal and specifically axon levels. This represents new evidence of the interesting correlation between cancer and neuronal pathways, especially the ones related to axonal growth and regeneration during injury. Further studies should elucidate the functional implications of those specific mRNAs regulated by PDCD4 in neurons, and particularly those acting locally in axons, a process that could provide novel functional insights in both plasticity and regenerative processes.

## MATERIALS AND METHODS

### Animals and injury procedures

Sprague-Dawley male adult rats (6–9 mo old) were used for ex vivo experiments. The maintenance was made in accordance with international agreements at IIBCE bioterium in Montevideo, Uruguay. Sciatic nerve transection was performed as in Canclini and collaborators (Canclini et al. 2014). All the experimental procedures were made according to the Uruguayan ethical national committee (CNEA) with approved project code “005/01/2014.”

### Cell cultures

Rats (Sprague-Dawley) and mice (C57/BL6) used for primary neuron cultures were housed at the Animal Unit in the School of Life Sciences (University of Nottingham). They were bred and sacrificed according to the UK Animal (Scientific Procedures) Act 1986. Primary cortical neuron cultures were obtained from C57/BL6 E16 mice brains as previously described (Lucci et al. 2020). Primary DRG cultures were obtained from E18 rat embryos. PC12 cell lines from ATCC (ATCC CRL-1721) were grown in collagen I (ThermoFisher, Cat# A1048301) coated plastic surfaces at 8 µg/cm^2^. Complete medium was made of RPMI (ThermoFisher, Cat# 31800022), 10% of horse serum (ThermoFisher, Cat# 26050088), 5% of fetal bovine serum (Capricorn Scientific, Cat# FBS-11A), and antibiotics (Sigma-Aldrich, Cat# A5955). The cells were cultured following commercial instructions and neuronal differentiation was achieved by removing growth factors and antibiotics and exposing cells to 100 ng/mL of NGF 2.5S (ThermoFisher, Cat# 13257019) for at least 72 h. Neuroblastoma (Neuro2a) cell lines were a kind gift from the Robert Layfield laboratory, University of Nottingham, UK. For more details about cell cultures, please see Supplemental Materials and Methods.

### siRNA and plasmid transfections

Primary cortical neurons and Neuro2a cells were both transfected 24 h after neuron seeding (day 2 of culture). For siRNA experiments we used 25 nM of siGENOME Mouse Pdcd4 SMARTpool 5 nmol (Cat# M-044032-01-0005) or siGENOME Non-Targeting siRNA Control Pool No.1, 5 nmol (Cat# D-001206-13-05), both from GE Healthcare Dharmacon—Horizon Solutions. Transfection protocols followed manufacturer's instructions using Lipofectamine 2000 (Invitrogen, ThermoFisher). Plasmid transfections were performed with 2 µg of PDCD4-pcDNA 3.1 (zeromycin), kindly gifted by Yang Hsin-Sheng, using an empty plasmid as control. Cortical neurons were cotransfected with 1 µg pmax-GFP Green-cat (ThermoFisher). In the specific case of primary DRG neuron cultures, the cell permeable Accell SMART POOL Pdcd4 siRNA 5 nmol (Cat# E-097927-00-0005) or Accell Non-Targeting Pool 5 nmol (Cat# D-001910-10-05), both from GE Healthcare Dharmacon—Horizon Solutions, were incubated at 1 µM final concentration in the cell body side of compartmentalized chambers after DRGs develop neurites. The PC12 cell line was transfected using commercial lentiviral particles from Dharmacon GE, with an inducible shRNA against PDCD4 (SMARTchoice Inducible Rat PDCD4 PGK–turboGFP shRNA, 100 μL, 10^7^ TU/mL; Material# VSR6432-223515627), or shScrambled control (SMARTchoice Inducible Non-targeting Control PGK/TurboGFP, 50 μL, 10^7^ TU/mL; Material# VSC6580). After transfection, cells were grown in complete medium for 24 h and selected with 5 µg/mL of puromycin (P7255, Sigma-Aldrich) for 3–5 d. Cells were then cultured in complete medium to obtain stable cell lines able to induce silencing of PDCD4 or express a scrambled shRNA control.

### Immunohistochemistry and immunocytochemistry

For tissue sections, rats were intracardially perfused with 3% sodium citrate (S4641-500, Sigma-Aldrich) and 4% PFA (158127, Sigma-Aldrich) in Phosphate Buffer Saline (PBS) buffer pH 7.4 (137 mM NaCl, 2.7 mM KCl, 8 mM Na_2_HPO_4_, and 2 mM KH2PO4). Following standard cryoprotection and 0.5% triton X-100 (13444259, ThermoFisher) permeabilization, 20 µm cryosections were made. Incubation with primary and secondary antibodies was performed in a blocking buffer with 5% NGS (MERCK, NS02L) overnight at 4°C. The PHEM buffer (60 mM PIPES, 25 mM HEPES, 10 mM EGTA, 2 mM MgCl_2_) was used for washes. For primary neurons and cell lines, cells were rinsed with PBS buffer and fixed with 4% PFA, 5 mM CaCl_2_ and 4% sucrose in PBS buffer for 30 min (RT), permeabilized in 0.2% Triton + 10 mM glycine in PBS for 20 min (RT) and incubated with antibodies overnight. Cells were mounted using Vectashield with DAPI (H-1200-10, VectorLabs) or Pro-Long Gold Antifade (P36930, ThermoFisher) mountant reagents. For a list of antibodies and probes used, please see Supplemental Material.

### Puro-PLA protocol

Ventral roots were extracted from adult rats and incubated in neurobasal media with puromycin at 300 µM. Then a fixation with 4% PFA for 1 h was performed and cryosections were made as described above. The PLA protocol was carried out according to the manufacturer's instructions of DuoLink, Sigma using the following reagents: Duolink In Situ PLA Probe Anti-Rabbit PLUS (Cat#DUO92002-30RXN), Duolink In Situ PLA Probe Anti-Mouse MINUS (Cat#DUO92004-30RXN), Duolink In Situ Detection Reagents FarRed (DUO92013-30RXN).

### Image acquisition and quantification analysis

Neocortex, cerebellum, sciatic nerves, and PC12 cell images were taken using an LSM confocal OLYMPUS FV300 with a 60× oil, NA 1.42 objective. For primary cultures, we used an inverted fluorescent microscope ZEISS axiovert 200 M coupled to a CCD camera (Photometrics CoolSnap MYO). For axonal length images, a 10× air NA 0.3 or 20× air NA 0.8 were used, while immunofluorescence quantification was done with a 63× oil NA 1.3. For PLA experiments on ventral roots, an LSM confocal ZEISS 800 was used with a 63× oil, NA 1.4. The stacks were always taken at an ideal μm number between each *z* plane.

For image quantification, the Fiji (*Just Image J*) tools (Schindelin et al. 2012) and the Neuron_Growth plugin software developed by Fanti and collaborators at the Universidad Nacional Autónoma de México (http://www.ifc.unam.mx/ffm/conditions.html) were used. For full details of quantification methods, see Supplemental Methods.

### Ribosome profiling

Cells were treated with 100 mg/µL of cycloheximide (01810, Sigma-Aldrich) for 1 h at 37°C in the hood to stop translation before collection of RNA on ice. A transcriptome sample was separated to use as total RNA control and submitted to RNA extraction using mirVana Isolation Kit (ThermoFisher, Cat# AM1560) and RNA-seq protocol. At the same time, a proteome sample was separated and submitted to label-free quantitative proteomics using an LC-MS/MS Orbitrap Fusion. For translatome samples, cells were lysed and a polysomal pellet was obtained by ultracentrifugation in sucrose cushion, resuspended and digested with Benzonase (Sigma-Aldrich, Cat# E1014). Ribosomal footprints were isolated running a denaturalized 15% PAGE 7 M urea, cutting the proper band identified by length (∼30 nt) and extracting RNA from gel slice. Ribosomal footprints quality and quantity was checked using 2100 Agilent Bioanalyzer Small RNA Kit and submitted to small RNA-seq protocol. Two biological replicates per condition (shPDCD4 and shScrambled) and per compartment (transcriptome, translatome and proteome) were obtained.

### Sequencing and bioinformatic analysis

All transcriptome and translatome samples were sequenced in BGI Tech Solutions. Transcriptome samples were sequenced using RNA-Seq Quantification Library (Normal Library: 2–10 µg) protocol, with poly(A)^+^ selection and 20 million paired-end (2 × 100 bp) reads obtained. Translatome samples were submitted to Small RNA Library (Low-Input Library: 0.2–1 µg) protocol and 40 million single-end reads were obtained. Sequence data is available at the NCBI Sequence Read Archive (SRA; https:// trace.ncbi.nlm.nih.gov/Traces/sra/) under BioProject ID PRJNA611824.

Sequences were mapped using *bowtie2* (Langmead and Salzberg 2012) versus curated mRNAs described in the mouse genome (available at NCBI ftp site). Read counts were estimated by *featureCounts* (Liao et al. 2014) and differential gene expression analysis between transcriptomes or translatomes was done using *edgeR* (Robinson et al. 2010*)*. Normalized counts were exported and translational efficiency was calculated and contrasted between conditions (shPDCD4 vs. shScrambled) using *Xtail* R package (Xiao et al. 2016). Gene lists analysis was performed using the online free tool *STRING* (Jensen et al. 2009) and in-house software (Radío S, Sotelo-Silviera JR, and Smircich P, in prep.; https://github.com/sradiouy/IdMiner).

For comparison between potential PDCD4 translational targets and axonal transcriptomes, we used published axonal RNA-seq data sets (Zappulo et al. 2017; Nijssen et al. 2018). Downloaded FASTQ files were mapped to the *Mus musculus* genome (GRCm38) with HISAT2 (Kim et al. 2015). StringTie (Pertea et al. 2015, 2016) was used to assemble and quantify the transcripts. For subsequent analysis, low expression genes were removed (TPM < 1). For the comparison, mouse orthologs of the potential PDCD4 translational targets were used, since all the axonal transcriptomes data sets come from murine models. The Venn diagram was performed with the VennDiagram package of R (Chen and Boutros 2011). The EASE Score (a modified Fisher exact *P*-value) was used to test if axonal transcriptomes were enriched in potential PDCD4 translational targets, and specifically those related with axonal growth. Extended protocols and details are available in SI Appendix.

## SUPPLEMENTAL MATERIAL

Supplemental material is available for this article.

## Supplementary Material

Supplemental Material
