# Supplementary material for: PDCD4 regulates axonal growth by translational repression of neurite growth-related genes and is modulated during nerve injury responses
Source: RNA. 2020 Nov;26(11):1637–53. doi: 10.1261/rna.075424.120 (PMC7566564; doi:10.1261/rna.075424.120)
Supplement: Supplemental Material [file supp_26_11_1637__index.html]

PDCD4 regulates axonal growth by translational repression of neurite growth-related genes and is modulated during nerve injury responses — Supplemental Material 

# PDCD4 regulates axonal growth by translational repression of neurite growth-related genes and is modulated during nerve injury responses

## Supplemental Material

- Supplemental\_Fig\_S1.pdf
- Supplemental\_Fig\_S2.pdf
- Supplemental\_Fig\_S3.pdf
- Supplemental\_Fig\_S4.pdf
- Supplemental\_Fig\_S5.pdf
- Supplemental\_Fig\_S6.pdf
- Supplemental\_Fig\_S7.pdf
- Supplemental\_Fig\_S8.pdf
- Supplemental\_Material.docx
- Supplemental\_Table\_S1.xlsx
- Supplemental\_Table\_S2.xlsx
