# Supplementary figures and images for: PDCD4 regulates axonal growth by translational repression of neurite growth-related genes and is modulated during nerve injury responses
Source: RNA. 2020 Nov;26(11):1637–53. doi: 10.1261/rna.075424.120 (PMC7566564; doi:10.1261/rna.075424.120)

**A**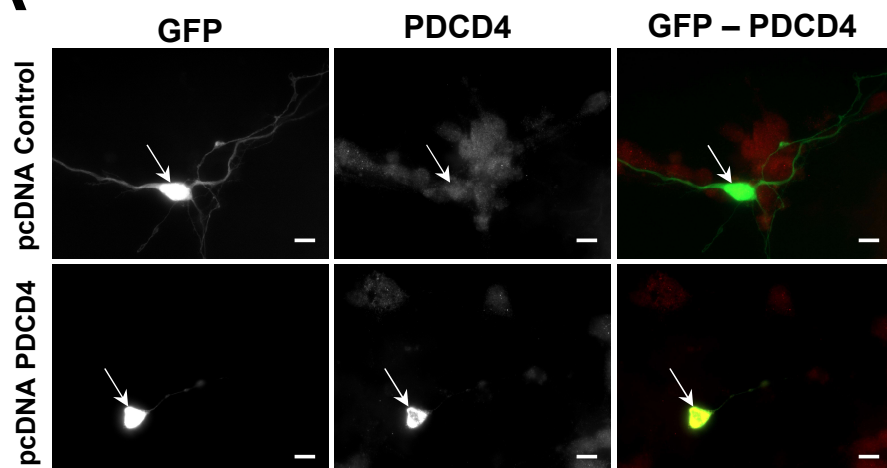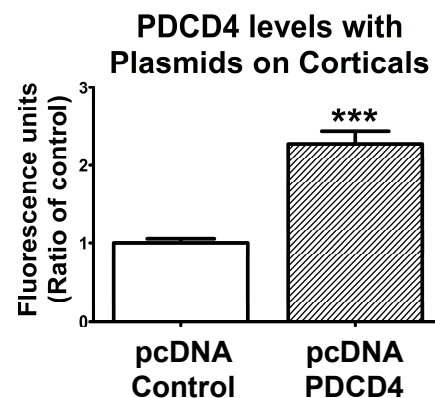**B**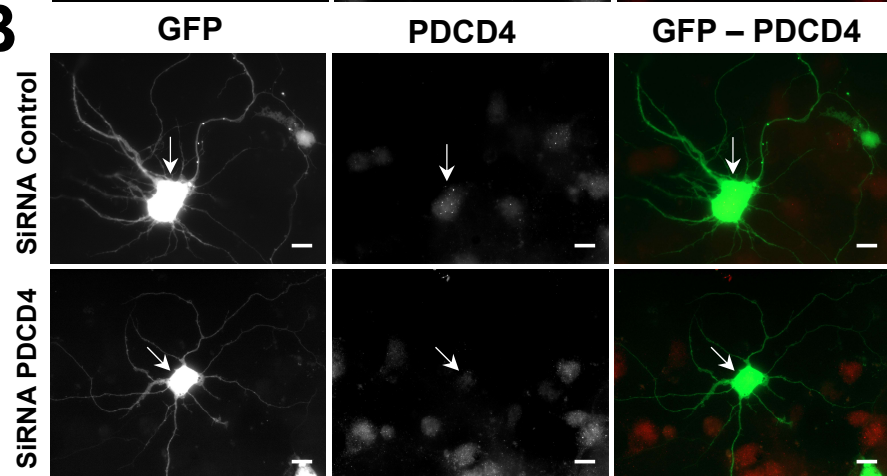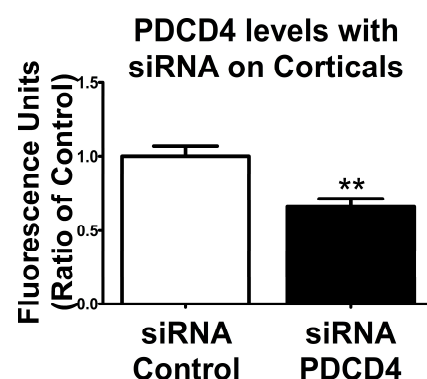**C**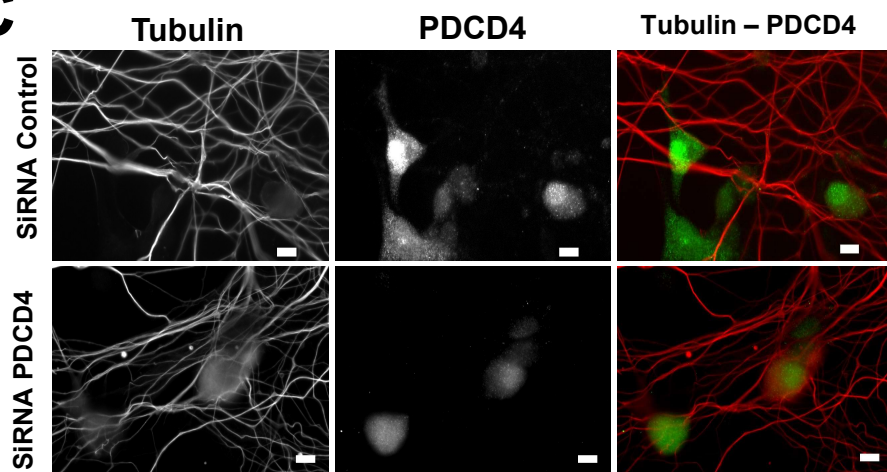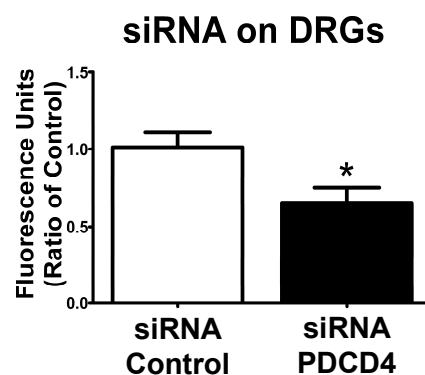**D**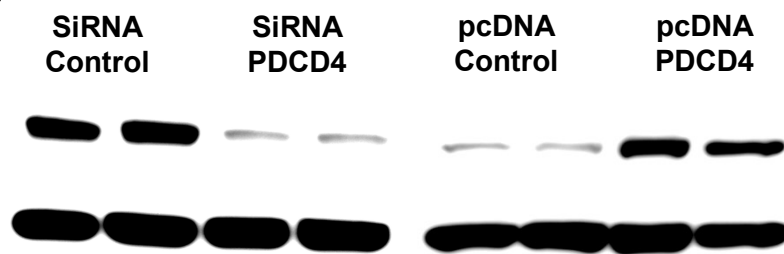

**Western Blot on N2A cell line**

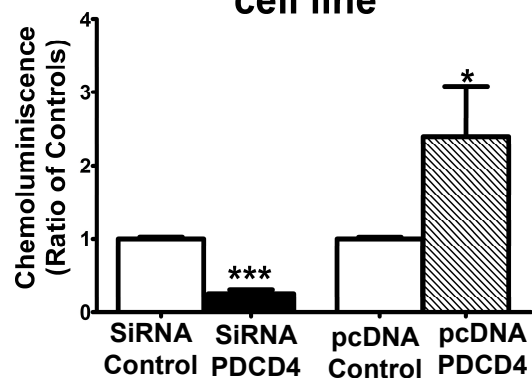

Supplement: Supplemental Material [file supp_075424.120_Supplemental_Fig_S1.pdf]

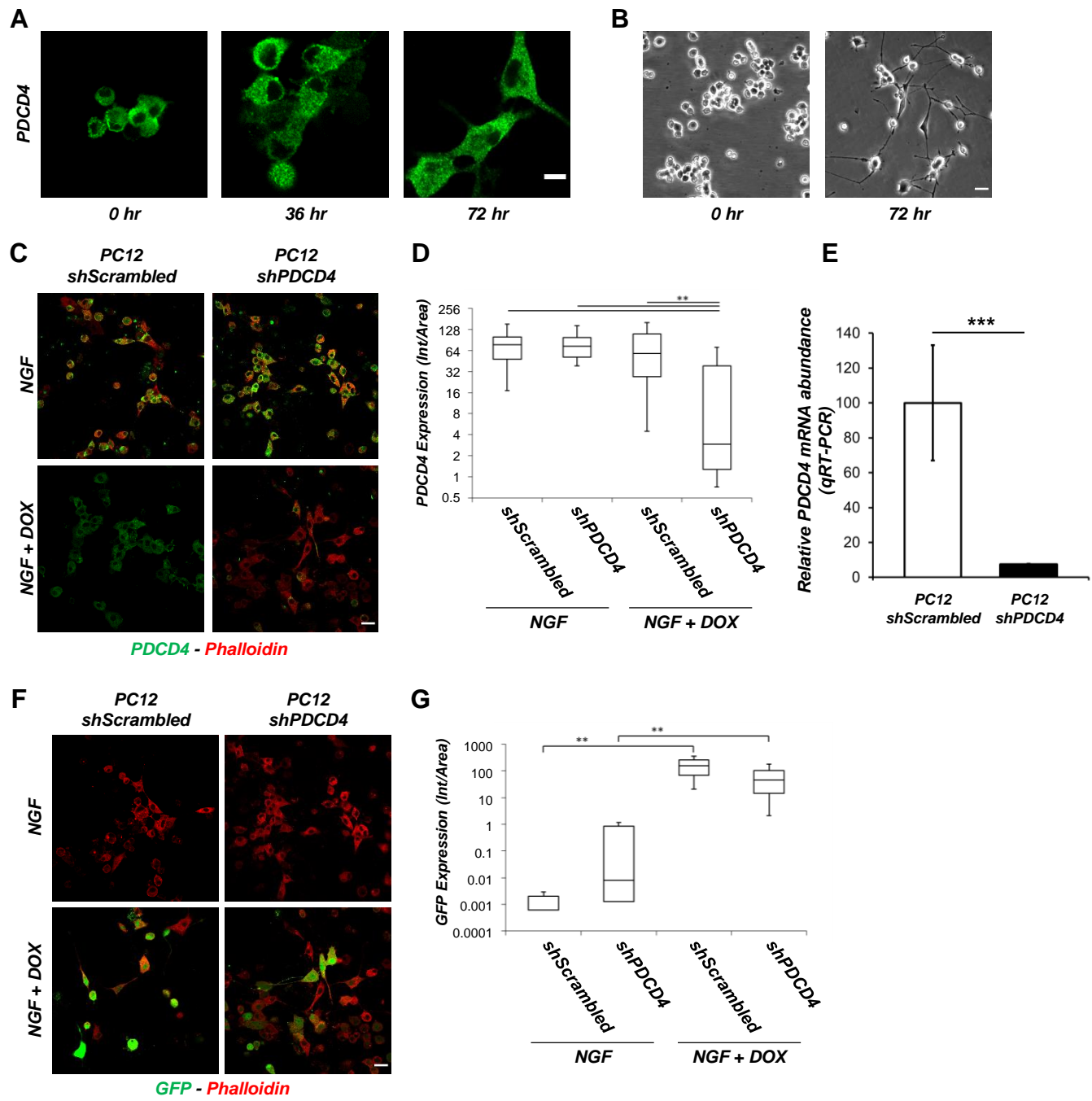

Supplement: Supplemental Material [file supp_075424.120_Supplemental_Fig_S2.pdf]

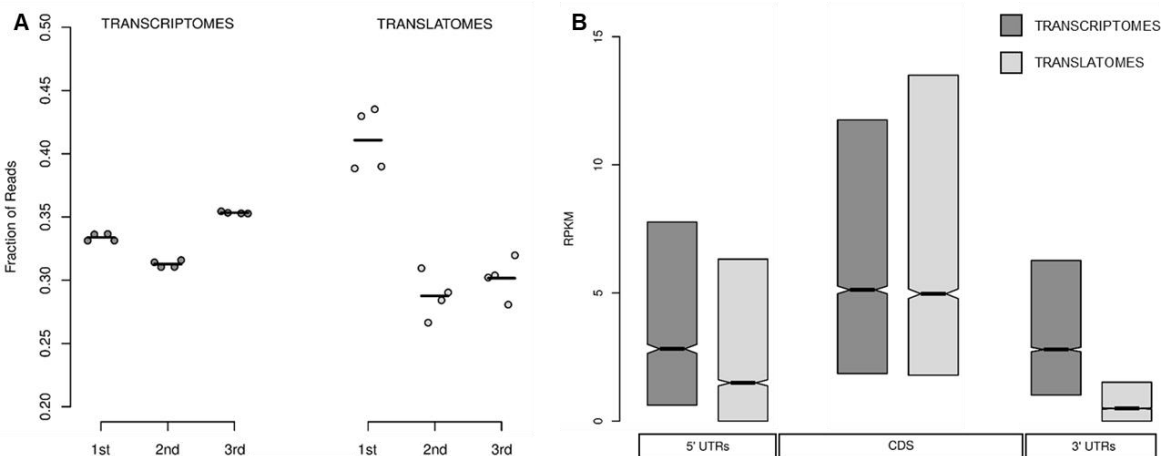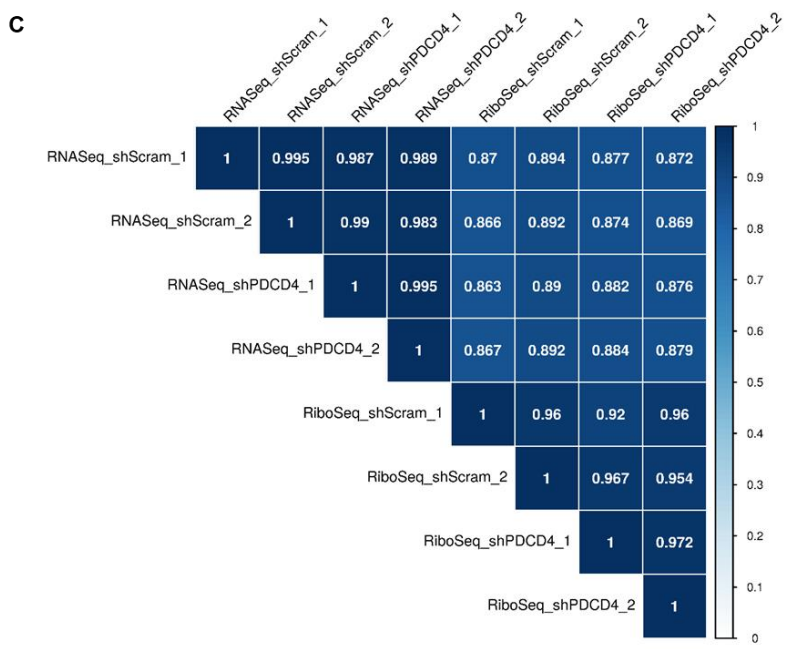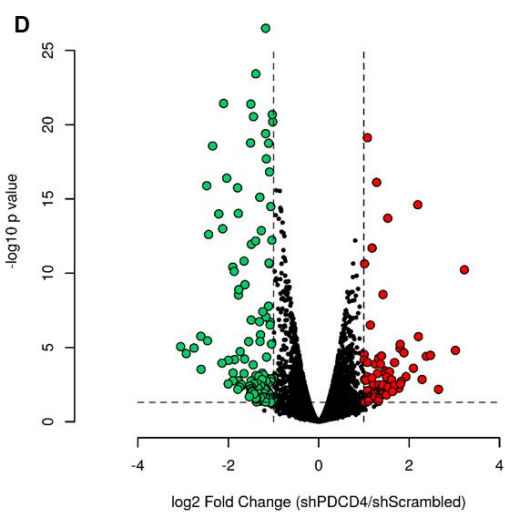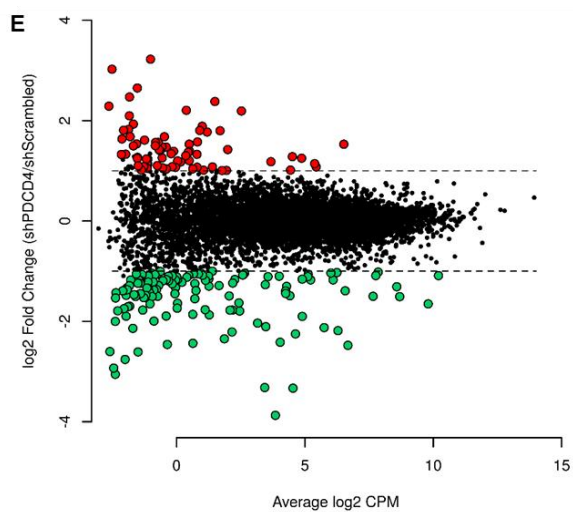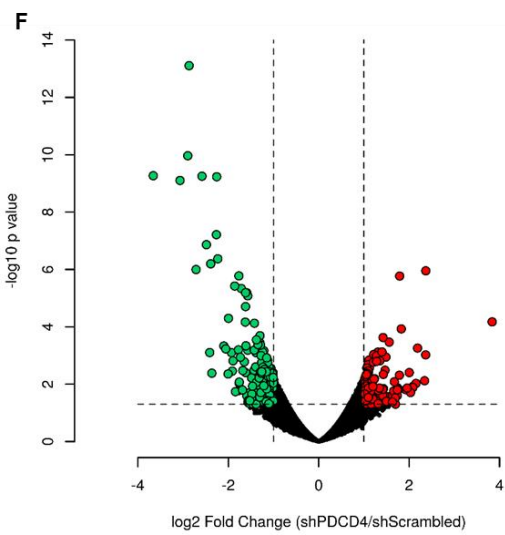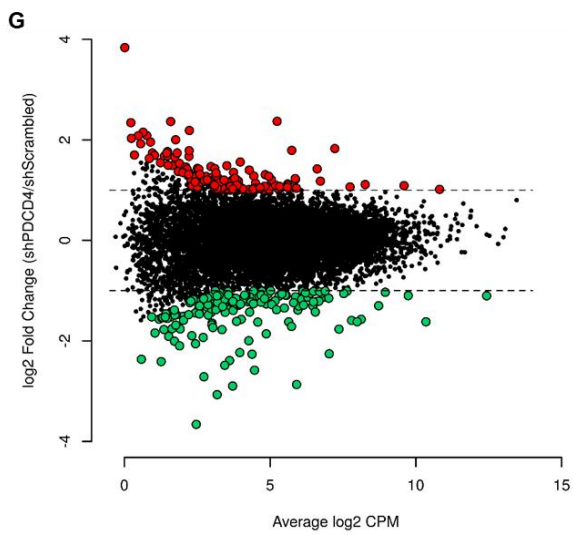

Supplement: Supplemental Material [file supp_075424.120_Supplemental_Fig_S3.pdf]

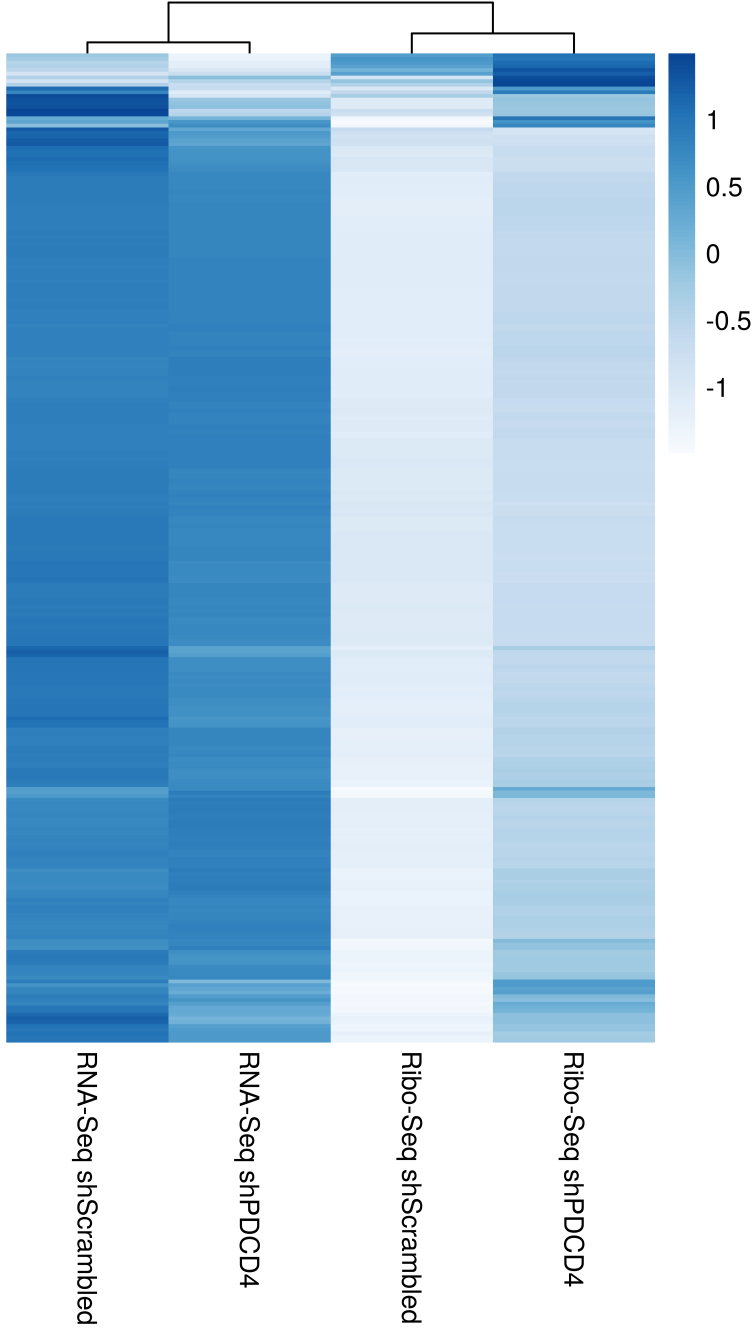

Supplement: Supplemental Material [file supp_075424.120_Supplemental_Fig_S4.pdf]

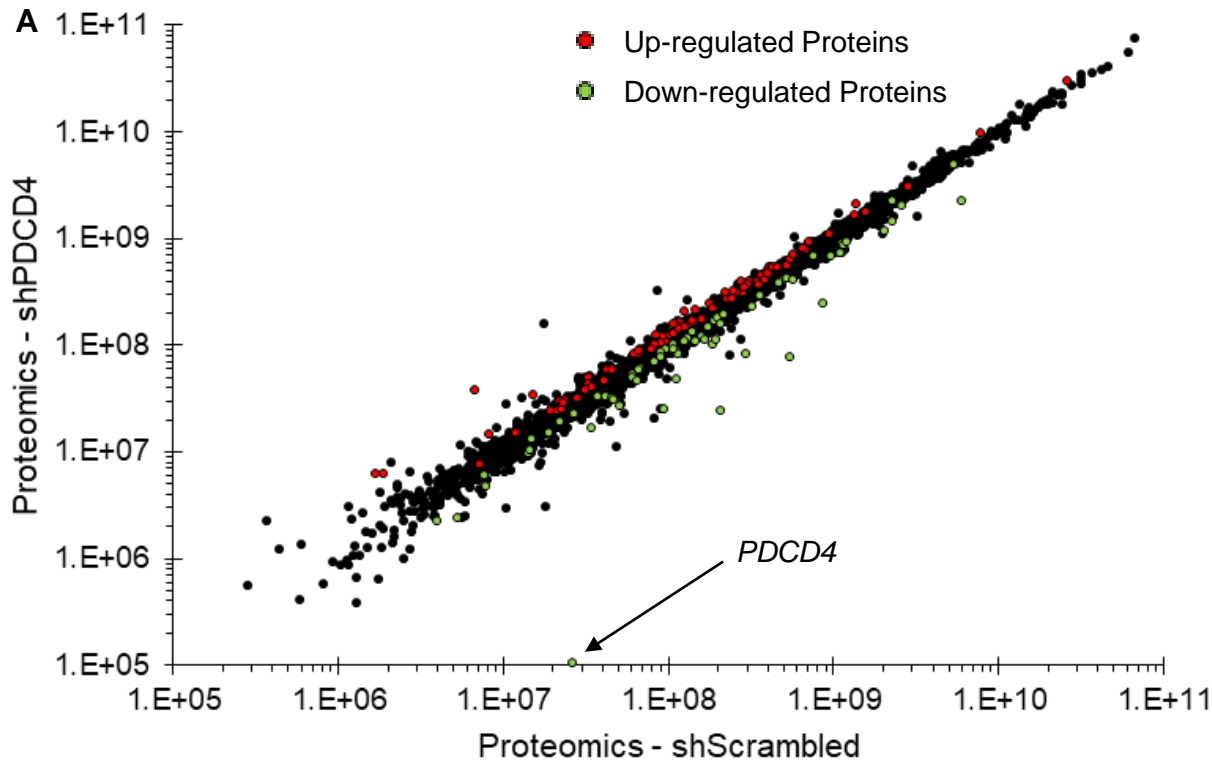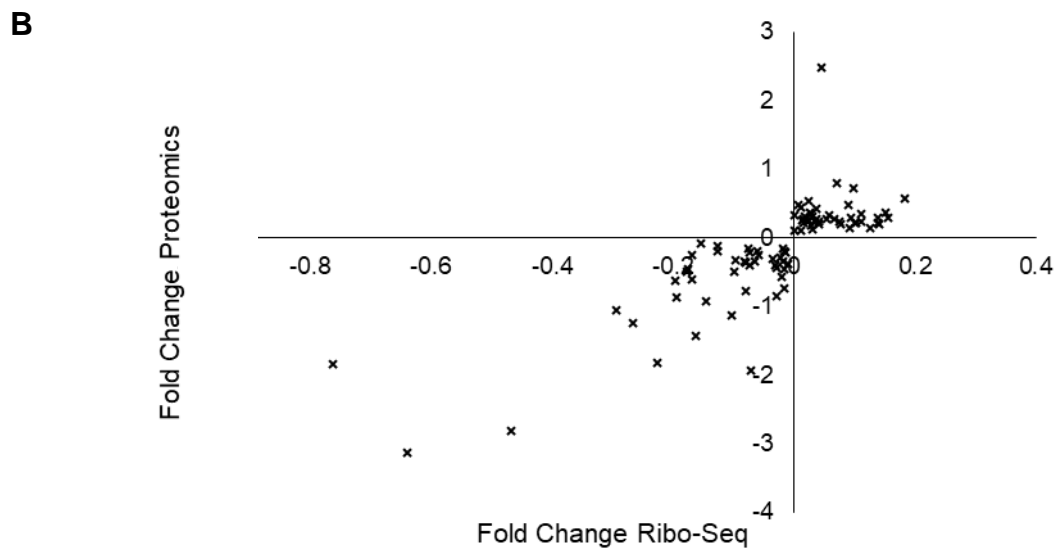

Supplement: Supplemental Material [file supp_075424.120_Supplemental_Fig_S5.pdf]

# B

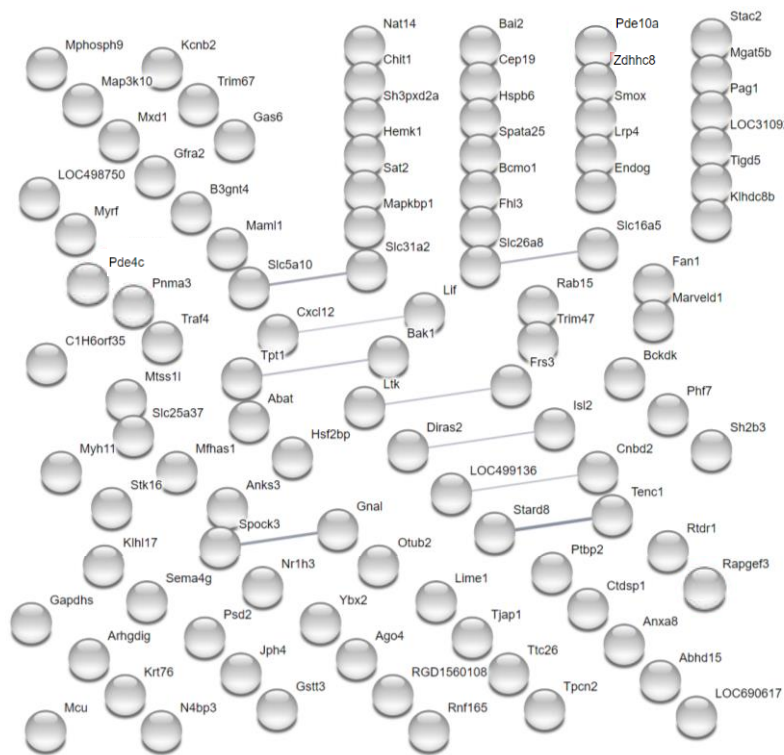

Supplement: Supplemental Material [file supp_075424.120_Supplemental_Fig_S6.pdf]

**A***Lentiviral particles shPDCD4**Lentiviral particles shPDCD4*

- + ++

- + ++

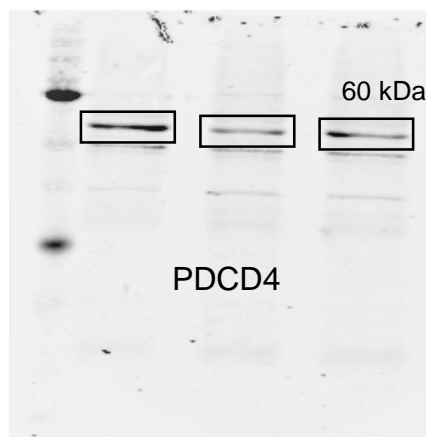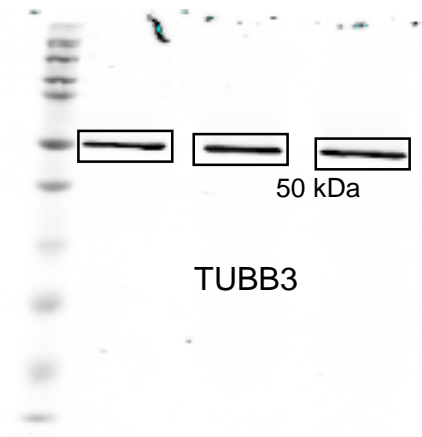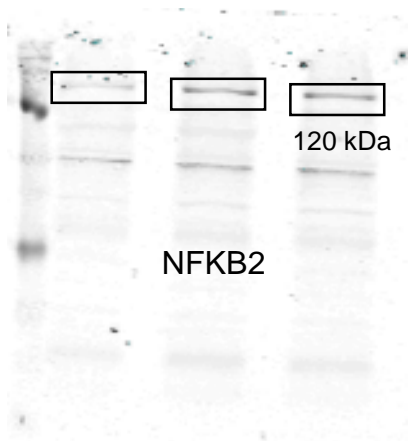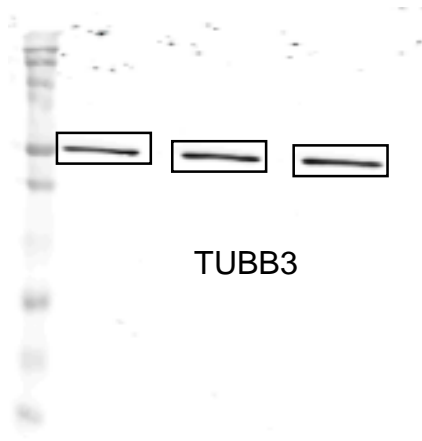**B**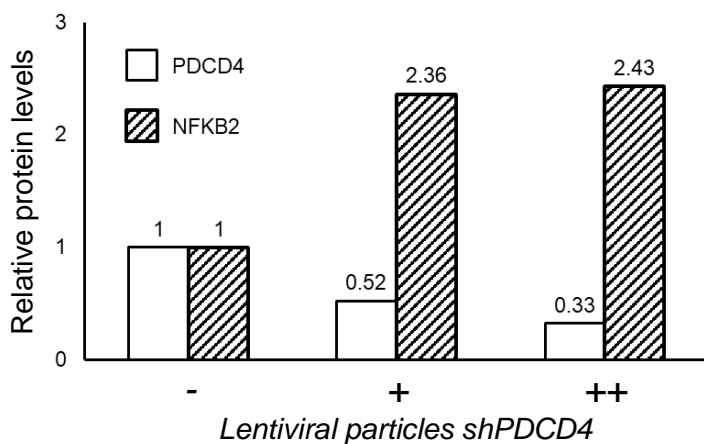

Supplement: Supplemental Material [file supp_075424.120_Supplemental_Fig_S7.pdf]
