# Supplementary material for: PDCD4 regulates axonal growth by translational repression of neurite growth-related genes and is modulated during nerve injury responses
Source: RNA. 2020 Nov;26(11):1637–53. doi: 10.1261/rna.075424.120 (PMC7566564; doi:10.1261/rna.075424.120)
Supplement: Supplemental Material [file supp_075424.120_Supplemental_Fig_S8.pdf]

A

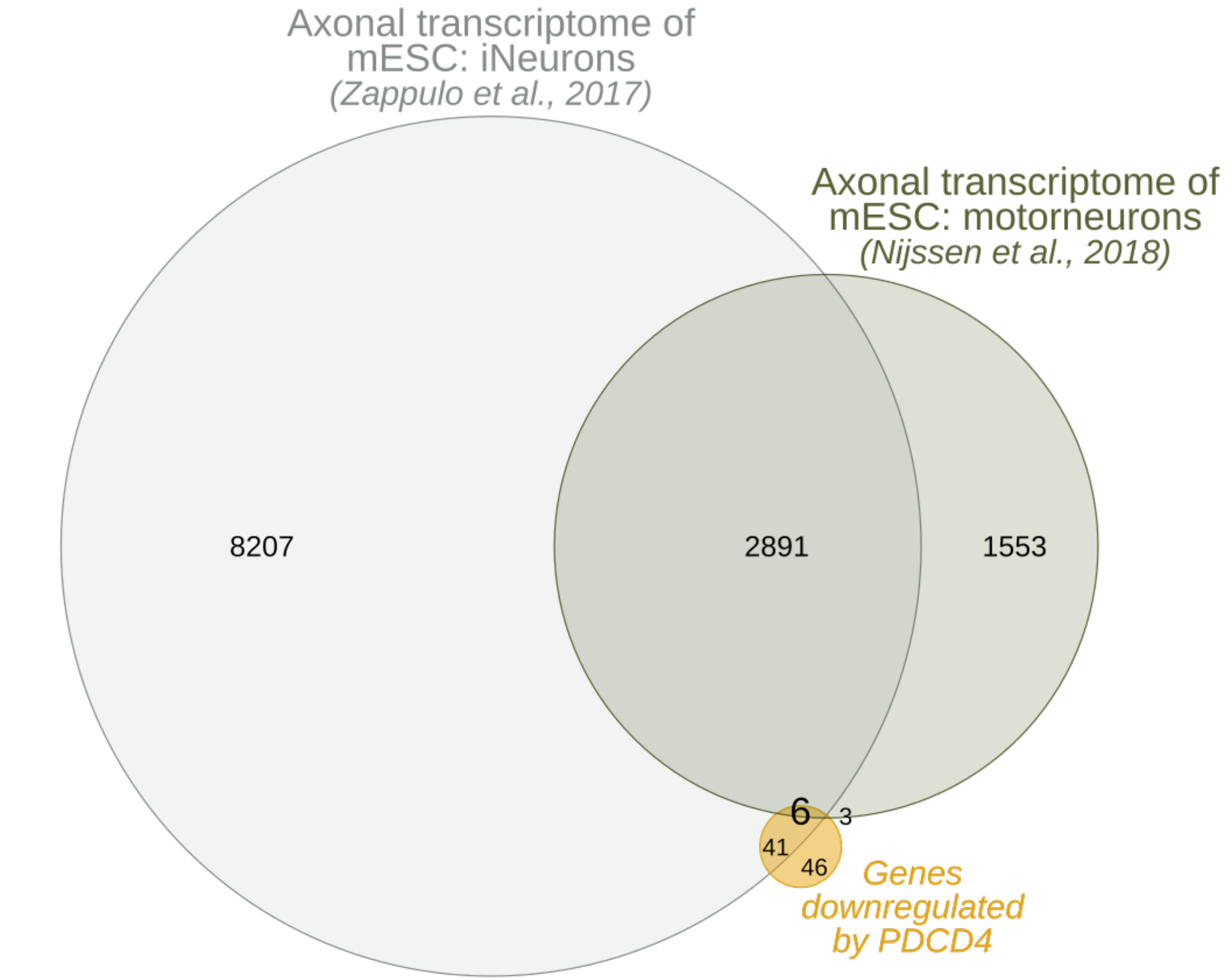

B

Neuron	Total genes	In common with genes downregulated by PDCD4	Reference
		All (EASE Score)	
mESC:iNerons	11145	47 (0.2263)	Zappulo *et al., 2017*
mESC:motorneuron	4453	9 (0.1118)	Nijssen *et al., 2018*
