# Supplementary material for: PDCD4 regulates axonal growth by translational repression of neurite growth-related genes and is modulated during nerve injury responses
Source: RNA. 2020 Nov;26(11):1637–53. doi: 10.1261/rna.075424.120 (PMC7566564; doi:10.1261/rna.075424.120)
Supplement: Supplemental Material [file supp_075424.120_Supplemental_Material.docx]

**Supplementary Information**

*PDCD4 regulates axonal growth by translational repression of neurite growth-related genes and is modulated during nerve injury responses*

Andrés Di Paolo, Guillermo Eastman, Raquel Mesquita-Ribeiro, Joaquina Farias, Andrew Macklin, Thomas Kislinger, Nancy Colburn, David Munroe, José Sotelo Sosa, Federico Dajas-Bailador, José Roberto Sotelo-Silveira.

This file contains:

- Supplementary Materials and Methods
- Supplementary Figures Legends
- Supplementary Tables Legends
- SI References

**Supplementary Materials and Methods**

*Animals and injury procedures*

Sprague Dawley male adult rats (6-9 months old) were used for ex vivo experiments. The maintenance was made in accordance with international agreements at IIBCE bioterium in Montevideo, Uruguay. Animals were anesthetized with ketamine and xylazine mix (100 mg/kg, 10 mg/kg) by intraperitoneal injection. Afterwards the animals were intracardially perfused using paraformaldehyde.

- Sciatic nerve transection

Adult Sprague-Dawley rats were anesthetized with 50 mg/kg pentobarbital. An incision was made at mid-thigh and the sciatic nerve was transected, which was then closed with cyanoacrylate glue. After 18 h recovery, rats were euthanized by decapitation and a 2-cm sciatic nerve segment proximal to the transection was removed. Equivalent contralateral uninjured segments were used as controls. Afterwards we labelled the proximal region using a strand and quickly fixed with PFA 4% for 1 hour at RT. Immunohistochemistry protocols were carried out as described for *in vivo* tissues. All the experimental procedures were made according to the Uruguayan ethical national committee (CNEA) with approved project code “005/01/2014”.

*Cell Cultures*

Rats (Sprague-Dawley) and mice (C57/BL6) used for primary neuron cultures were housed at the Animal Unit in the School of Life Sciences (University of Nottingham). They were bred and sacrificed according to the UK Animal (Scientific Procedures) Act 1986.

- Primary cortical neurons culture

Primary cortical neuron cultures were obtained from C57/BL6 E16 mice brains as previously described [(Lucci et al. 2020)](https://paperpile.com/c/bm7mcc/ALjj). Brain cortices were dissected, and the meninges separated under a dissection microscope. The tissue was further incubated in Hanks Balanced Salt Solution (HBSS, Ca^2+^ and Mg^2+^-free; Gibco) with 1 mg/ml trypsin and 5 mg/ml DNAse I (Sigma-Aldrich) at 37°C for 30’. Following the addition of 0.05% (v/v) soybean trypsin inhibitor (Sigma-Aldrich), the tissue was mechanically dissociated in Neurobasal media (Invitrogen) supplemented with 1X GlutaMax and 2% B-27 (Gibco). Dissociated neurons were resuspended in supplemented Neurobasal media (10x10^6^ cells/mL).

- Primary DRG neurons culture

Primary DRGs cultures were obtained from E18 rat embryos. Briefly, approximately 200 DRGs ganglions were extracted from 11-15 embryos per experiment in L15 cold media, trypsinized in Ca^2+^-Mg^2+^ PBS 0.025% trypsin for 10 minutes at 37°C, 5% CO_2_ and incubated with 0.1% collagenase type I (filtered 0.2 µm) for 20 min, 37°C, 5% CO_2_. Pellet is resuspended and dissociated in DMEM *complete* media (2% B27, 2 mM glutamate, 1% PE, 50 ng/mL NGF, 50 ng/µL GDNF, 4 µM APH). Cells were centrifuged at 1000 g for 5 minutes, and the pellet resuspended in complete media before seeding into compartmentalized microfluidic chambers (Xona Microfluidics, Xona SND 150) pre-coated with laminin 20 µg/mL for 1 hour, 37°C. Then 10 µL of dissociated DRGs were seeded into the designated channel (approximately 30 DRGs in each).

- PC12 cell line culture and lentiviral transfection

PC12 cells from ATCC were grown in RPMI 1640 medium (Gibco) supplemented with 10% horse serum, 5% of fetal bovine serum and antibiotics (penicillin and streptomycin), at 37°C with 5% CO_2_. Plastic surfaces were coated with 8 µg/cm^2^ collagen I from rat tail. Neuronal differentiation was achieved by removing growth factors and antibiotics and exposing cells to 100 ng/mL of NGF 2.5S for at least 72 h. To achieve PDCD4 silencing, commercial lentiviral viral particles from Dharmacon GE, with an inducible shRNA against PDCD4, were used. After transfection, cells were grown in complete medium for 24 hrs and selected with 5 µg/mL of puromycin for 3-5 days. Cells were then cultured in complete medium to obtain stable cell lines able to induce silencing of PDCD4 or express a scrambled shRNA control.

- Neuroblastoma (Neuro2a) Cell line culture

Neuro2a cells were a kind gift from Robert Layfield lab, University of Nottingham, UK. Cells were maintained in DMEM, 10% FCS, 1% PS and seeded on 12-well plates at 1.5x10^5^ cells/well.

*siRNA and plasmid transfections*

In the primary cortical neuron and Neuro2a cell line, transfections were performed 24 hours after neuron seeding (day 2 of cell culture) using Lipofectamine 2000 (Invitrogen) as suggested by manufacturer’s instructions. 25 nM of siGENOME Mouse Pdcd4 SMARTpool 5 nmol (catalog number M-044032-01-0005) and siGENOME Non-Targeting siRNA Control Pool N°1 5 nmol (catalog number D-001206-13-05) both from GE Healthcare Dharmacon-Horyzon Solutions, using Lipofectamine 2000 (Invitrogen, Thermo Scientific), following the manufacturer's instructions. For plasmid transfections the PDCD4-pcDNA 3.1 (zero) was kindly gifted by Yang Hsin-Sheng, with empty plasmid used as a control. Both plasmids were applied at 2 µg final concentration and added 24 hours after neuron seeding (day 2 of cell culture). To identify transfected cortical neurons the siRNA and PDCD4 plasmids were co-transfected with pmax-GFP Green-cat (ThermoScientific) 1 µg of final concentration and cells were fixed 72 hours after transfection (day 5 of cell culture). In the specific case of primary DRG neuron cultures, the cell permeable Accell SMARTPOOL Pdcd4 siRNA 5 nmol (catalog number E-097927-00-0005) or Accell Non Targeting Pool 5 nmol (catalog number D-001910-10-05) both from Healthcare Dharmacon - Horyzon Solutions were incubated at 1 µM final concentration in the cell body side of compartmentalized chambers after DRGs develop neurites. 96 hours later neurons were fixed.

*Semi-quantitative Real Time PCR*

PDCD4 quantification in PC12 cells was performed by semi-quantitative real time PCR and ΔΔCt method [(Livak and Schmittgen 2001)](https://paperpile.com/c/bm7mcc/ycMr). For this, RNA was isolated using Trizol (Invitrogen, Cat#15596026) or mirVana isolation kit (ThermoFisher, Cat# AM1560). Genomic DNA was removed by DNAse treatment (Invitrogen, Cat#AM2222) and RNA was re-isolated by the same method. cDNA was produced by retrotranscription by SuperScript II (Invitrogen, Cat#18064014) or III (Invitrogen, Cat#18080044) reverse transcriptase and quantified with SYBR Green Master Mix (Applied Biosystems, Cat#A25742) in Corbett Rotor-Gene 6000 instrument with the following PCR conditions: 10 minutes at 95°C, 40 cycles of: 15 seconds at 95°C, 15 seconds at 58°C and 45 seconds at 60°C; and 72°C to 90°C melting. In all cases, three independent biological replicates were used to evaluate significant differences by Student’s test using RPL29 gene as housekeeping [(Zhou et al. 2010)](https://paperpile.com/c/bm7mcc/vzSr).

For primary DRGs cultures, E18 rat embryos were cultured at two channel compartmentalized microfluidic chambers as described in the previous section. Axonal RNA was obtained as described previously [(Garcez et al. 2016)](https://paperpile.com/c/bm7mcc/rJ5Y). To obtain cDNA, 50 ng of RNA were retrotranscribed by Superscript III reverse transcriptase (ThermoFisher, Cat#18080093) according to manufacturer instructions. The qPCR was performed using PowerUp SYBR Green (Applied Biosystems) in an Applied Biosystems Step One Plus thermocycler, using cycling parameters recommended by Applied Biosystems (mRNA). Data was acquired with Applied Biosystems SDS2.3 software. As above, RPL-29 gene was used as housekeeping gene [(Zhou et al. 2010)](https://paperpile.com/c/bm7mcc/vzSr).

Primers sequences were obtained from literature: PDCD4 forward: 5'-TGAGCACGGAGATACGAACGA-3' and PDCD4 reverse: 5'-GCTAAGGACACTGCCAACACG-3' from [(Liu et al. 2010)](https://paperpile.com/c/bm7mcc/o2vu), RPL29 forward: 5'-CAAGTCCAAGAACCACACCAC-3' and RPL29 reverse: 5'-GCAAAGCGCATGTTCCTCAG-3' from [(Parker et al. 2013)](https://paperpile.com/c/bm7mcc/9Dho).

*Immunohistochemistry and immunocytochemistry*

- Tissue sections

For brain, cerebellum and sciatic nerve tissue, rats were intracardially perfused with 3% sodium citrate and then 4% PFA in PBS buffer. Tissue extraction was done after two hours of perfusion, and a final fixation step was performed overnight (ON), 4°C for brain and cerebellum and 1 hour at RT for sciatic nerves. 3x10 minutes washes in PBS and cryo-protection steps with successive incubations in 15 and 30% sucrose ON, 4°C for one and two days respectively were performed. Cryosections of 20 µm were collected on glass slides pre-treated with Poly-L-Lysine 0.1% in mQ water. Permeabilization with 0.5% Triton in the PHEM buffer for 20 minutes at RT, 3x5 washes in PHEM buffer and blocking in 3% BSA, 1% glycine for 30 minutes at RT were developed. Incubation with the primary and secondary antibodies was performed in a blocking buffer with 5% NGS, ON at 4°C with 3x10 minutes washes in buffer PHEM between and after incubation.

- Primary neurons and cell lines

For cortical, DRG neurons, PC12 and Neuro2a cell lines, the media was removed, and cells rinsed with PBS and fixed with 4% PFA with 5 mM CaCl_2_ and 4% sucrose in PBS buffer for 30 minutes (RT). Cells were permeabilized in 0.2% Triton + 10 mM glycine in PBS for 20 minutes at RT. Incubation with the primary antibody was made in 3% BSA ON at 4°C, while incubation with secondary antibodies was made in the same buffer for 2 hours at RT. Cells were mounted using Vectashield with DAPI or Pro-Long Antifade reagent.

*List of antibodies and probes*

Rabbit polyclonal PDCD4 antibody (dilution 1:200) Abcam (ab51495); Mouse monoclonal MAG antibody (dilution 1:1500) Millipore (Cat#MAB1567); Mouse monoclonal antibody Phospho-p70 S6 Kinase (Thr389) (1A5) (dilution 1:400) CellSignaling (#9206); Mouse Monoclonal Anti-Acetylated Tubulin antibody (dilution (1:1000) Sigma - Aldrich (Cat#T7451); Alexa Fluor 555 Phalloidin (dilution 1:150) Invitrogen-ThermoFisher (Cat#A34055); Alexa Fluor 647 Phalloidin (dilution 1:150) Invitrogen-ThermoFisher (Cat#A22287); Goat anti-Mouse IgG, (H+L) HRP conjugate antibody Millipore (Cat# AP308P); Swine anti-rabbit IgG HRP antibody Dako (Cat# P0217); Goat anti-Mouse IgG (H+L) Cross-Adsorbed Secondary Antibody Alexa Fluor 488 (dilution 1:1000) ThermoFisher (Cat# A-11001); Goat anti-Mouse IgG (H+L) Secondary Antibody, Alexa Fluor 555 conjugate (dilution 1:1000) ThermoFisher (Cat# A-21422); Goat Anti-Mouse IgG (H+L) Antibody Alexa Fluor 633 Conjugated (dilution: 1:1000) ThermoFisher (Cat# A-21052); DAPI (dilution 1:2000) ThermoFisher (Cat#D1306).

*Puro-PLA protocol*

A total of 2 independent experiments were made. Ventral roots were extracted from adult rats and incubated in neurobasal media with puromycin at 300 µM final concentration for 15 and 30 minutes or without puromycin as a control condition. Then a fixation with 4% PFA for 1 hour was performed and cryosections were made as described above.

The PLA protocol was carried-out according to the manufacturer's instructions of DuoLink, Sigma using the following reagents: Duolink® In Situ PLA® Probe Anti-Rabbit PLUS dilution 1/5 (Cat#DUO92002-30RXN), Duolink® In Situ PLA® Probe Anti-Mouse MINUS dilution 1/5 (Cat#DUO92004-30RXN), Duolink® In Situ Detection Reagents FarRed (DUO92013-30RXN).

*Protein extraction and western blot protocol for Neuro2a cell line*

A total of 3 independent experiments were performed. Cells were scraped 72 h after transfection, lysed with 150 µL of RIPA (50 mM Tris-HCl, pH 8.0, with 150 mM sodium chloride, 1.0% Igepal CA-630 (NP-40), 0.5% sodium deoxycholate and 0.1% sodium dodecyl sulphate), centrifuged for 20 min at 12,000g and supernatants collected and run on a 12% SDS-PAGE gel. Proteins were separated by standard electrophoresis protocol. For blocking step 5% milk in TBST (TBS buffer with Tween-20 0.1%) was used for 1 hour at RT. The primary antibodies were incubated ON, 4°C and a chemiluminescence protocol was performed using HRP secondary antibodies. The enzyme substrate was the Supersignal West femto-maximum sensitivity substrate revealed and was incubated for 1 minute and then washed with blocking buffer. Western blot bands were detected with a Las 3000 mini from FUJIFILM using the accumulated signal method.

*Image acquisition*

Neo-cortex, cerebellum, sciatic nerves and PC12 cells images were taken on the LSM confocal OLYMPUS FV300 using a 60X oil, NA 1.42 objective. The software for controlling the microscope and taking the images was the Fluoview version 5.0c.

For primary cultures, an inverted fluorescent microscope ZEISS axiovert 200M coupled to a CCD camera (Photometrics CoolSnap MYO) was used. For axon length images, a 10X air NA 0.3 or 20X air NA 0.8 were used, while quantification of immunofluorescence used a 63X oil NA 1.3. The micromanager software version 1.4 was employed to acquire the images and control the microscope.

For PLA experiments on ventral roots an LSM confocal ZEISS 800 was used. A 63x oil, NA 1.4 premium objective adapted for AiryScan was used for image acquisition. The stacks were always taken at ideal μm number between each z plane. The software for controlling the microscope and taking the images was the ZEN Blue version 2.3.

*Image quantification and analysis*

In all image quantification analysis, we kept the same PMT (below 700 V) and/or time exposures of the camera in order to compare control and experimental conditions. We also kept the same PMT (below 700 V) and same laser intensities for each experiment between conditions, with all the images from each experimental group taken on the same day.

- Quantification of fluorescence

Quantification of expression levels from immunofluorescence signals at different regions of interest (ROIs) was made with Fiji (Just ImageJ) software [(Schindelin et al. 2012)](https://paperpile.com/c/bm7mcc/9xVRz). Following the selection of ROIs, the Raw_Integrated_Density was calculated and divided by the size of the selected area. For each condition an average of these values was calculated and normalized against its control. The value for the controls was defined as 1. For the selection of ROIs in cortical and DRG neurons the acetylated tubulin was used to define subcellular compartments. For quantitative analysis in cortical neurons a total of 292 cell bodies and 129 axons were quantified from three independent experiments (unless as indicated for day 12) In experiments with sciatic nerve ventral roots axoplasms, MAG, phalloidin and light-chain neurofilament 68 KDa were used as counter-stains.

- Quantification of axonal length of cortical neurons

Images were taken from at least four independent preparations. GFP-positive neurons were assessed with axons defined as a neurite that was longer than 80 μm and at least three times the length of other processes, measured from the cell body to the distal extent of the central region of the growth cone. Selected axon projections were measured using ImageJ, and the data expressed as percentage of the respective controls. For determination of axon versus branch, the axon was defined as the process that remained parallel to the axon segment proximal to the branch point. Branches were defined as processes extending at orthogonal angles to the axon. Total length of axon branches was not included in the measurement. The average length of axons in control groups was ± 341 μm (mean ± 14.92). The average for each experimental condition (siRNA PDCD4 or plasmid PDCD4) was normalized against its control. A total number of ~400 cortical axons were measured per condition for the PDCD4 overexpression analysis and ~600 axons per condition for the PDCD4 silencing analysis. The n number was defined as separate experiments from independent neuronal preparations for all conditions.

- Quantification of axonal length of DRG neurons

A stitched image of the entire axonal channel of the microfluidic chambers stained for axons (acetylated tubulin) was obtained using micromanager software and the stitching plugin of Fiji software for each axonal microfluidic compartment. A total of 4 independent experiments with siRNA control chambers and 3 with siRNA of Pdcd4 were used (in total 9 siRNA Control and 7 siRNA PDCD4 chambers were used). Image analysis for neuron growth was processed using the Neuron_Growth plugin software developed by Fanti & collaborators at the Universidad Nacional Autónoma de México (http://www.ifc.unam.mx/ffm/conditions.html). The same software settings were used for all images and the percentage of the area covered by the axons was quantified. Average values were calculated for the siRNA control chambers and for the siRNA PDCD4 chambers and normalized to the internal control.

- Quantification of neurite length of differentiated PC12 cells

Images were taken from five independent preparations. For quantifying neurite length of differentiated PC12 cells in the presence and absence of PDCD4, three independent cell cultures were growth for each condition. After 72 h of shRNA induction, cells were exposed to NGF for 72 h to achieve neuron-like differentiation. The free line tool of ImageJ was used to mark neurites in several optic fields for each culture and length was quantified. An average of 80 neurites were quantified per replicate. Neurite length was contrasted in the two conditions comparing 3 vs 3 averages per replicate

- Puro-PLA Pdcd4 spots quantifications

Fiji (just ImageJ) software was used to quantify PLA signal. After selection of axoplasm regions (counterstained with phalloidin and/or light-chain-neurofilament) from Z-stack images a z project with maximum intensity projection algorithm was used to obtain a single image with all the PLA spots for each stack. Then a threshold was defined to detect each spot as black and the rest as white. Then the “analyze particles” algorithm was used with the size between 0.20-10 in all cases. The “masks display exclude clear” algorithm was used to count and see the mask on each spot and to control if the protocol was adequately working for each image. The experiment without puromycin was used as a control condition and the count of PLA/area were defined as “1”. Following this, the PLA counts for 15 minutes and 30 minutes exposure to puromycin were normalized to the control.

- Western Blot quantification

Images were obtained with the same exposure settings. Fiji (Just ImageJ) gel quantification software was used selecting ROIs equal to the biggest protein band for each image. Each band was quantified and then divided by the one corresponding to actin (control band) for each line. The values corresponding to siRNA PDCD4 were expressed as a percent of the siRNA control.

*Ribosome Profiling*

After 72 h of 100 ng/mL NGF 2.5S and 500 ng/mL doxycycline exposure, to achieve neuronal differentiation and shRNA expression, 80x10^6^ cells per condition were used to isolate total RNA and polysomes, in two biological replicates per condition. For this, cells were treated with 100 mg/µL of cycloheximide (CHX) for 1 hour at 37°C in the hood to stop translation before collection of RNA on ice. A Transcriptome sample was separated to use as total RNA control and submitted to RNA extraction using mirVana isolation kit (ThermoFisher, Cat# AM1560) and RNA-Seq protocol. At the same time, a proteome sample was separated and submitted to label-free quantitative proteomics using an Orbitrap Fusion. For translatome samples, cells were lysed and post mitochondrial supernatant was loaded in a 12%-33.5% sucrose cushion and ultracentrifugated in SW40Ti rotor at 35,000 RPM for 2:45 hrs at 4°C. Polysomal pellet was resuspended and digested with 200 U of Benzonase for 10 minutes at RT. Digestion was stopped with mirVana Lysis Buffer to continue with RNA isolation. Ribosomal footprints were isolated running a denaturalized 15% PAGE 7M urea, cutting the proper band identified by length (~30nt) and extracting RNA from gel slice. Ribosomal footprints (Translatome sample) are then concentrated by precipitation and quality and quantity checked using 2100 Agilent Bioanalyzer Small RNA Kit.

*Sequencing and bioinformatic analysis*

All transcriptome and translatome samples were sequenced in BGI Tech Solutions. Transcriptome samples were submitted to RNA-Seq Quantification Library (Normal Library: 2-10 μg) protocol, using poly(A)+ selection and 20 millions of paired-end (2x100 bp) reads were obtained. Translatome samples were submitted to Small RNA Library (Low-Input Library: 0.2-1 μg) protocol and 40 millions of single-end reads were obtained.

Fastq files obtained were analyzed using servers available in the Genomics Department at IIBCE. Sequences were mapped using bowtie2 [(Langmead and Salzberg 2012)](https://paperpile.com/c/bm7mcc/bpZ1R) versus curated mRNAs described in the mouse genome (available at NCBI ftp site). For translatome samples, sequences were first mapped against *Rattus norvegicus* rRNA genes to remove contamination. Reads counts were estimated by featureCounts [(Liao et al. 2014)](https://paperpile.com/c/bm7mcc/c4WcF) and differential gene expression analysis between transcriptomes or translatomes was done using edgeR [(Robinson et al. 2010)](https://paperpile.com/c/bm7mcc/ezyHC). Normalized counts were exported and translational efficiency was calculated and contrasted between conditions (shPDCD4 vs shScrambled) using Xtail R package [(Xiao et al. 2016)](https://paperpile.com/c/bm7mcc/q9v5z). Gene lists analysis were performed using on-line free tools like STRING [(Jensen et al. 2009)](https://paperpile.com/c/bm7mcc/Bx1R7) and an in-house software (manuscript in preparation; <https://github.com/sradiouy/IdMiner>).

Protein samples were submitted to label-free quantitative proteomics using an LC-MS/MS Orbitrap Fusion instrument. Protein intensity values (LFQ/iBAQ) were averaged and statistical differences were reported using ANOVA.

*General statistical analysis*

All data groups shown are expressed as the mean ± SEM and the probability distribution of the data set was analyzed before further statistical analysis. Normality tests were made for all the data sets. With normal data, a two tailed t-student test was performed or paired t test for plasmid and siRNA axonal length quantifications. If the data sets don't pass the normality tests, a Mann Whitney test was used. In all cases p values less or equal to 0.05 were considered as significant. Each “n” was defined as an independent experiment from a separate culture preparation. The following general code was used: (*): p value <0.05; (**): p value <0.01; (***): p value <0.001.

**Supplementary Figures Legends.**

**Figure S1.** **(A)** Fluorescent image of cortical neurons overexpressing PDCD4: GFP and PDCD4 expression is shown in cortical neurons transfected with a PDCD4 cDNA plasmid or with a pcDNA control plasmid. In both cases, a GFP co-transfected plasmid was used as a positive evidence of transfection. White arrows indicate the transfected neurons (scale bar 20 µm). The right panel shows quantification of PDCD4 levels, detecting an increase of 2.3 fold compared to the control condition (p value (***) ≤0.001, two tailed Mann Whitney test, n=3 with 3 technical replicates, error bars: SEM). **(B)** Same as (A) but for cortical neurons were PDCD4 was knockdown using a mix of 4 siRNA sequences against PDCD4 (Dharmacon siRNA SMARTPOOL) or a mix of 4 scrambled siRNA sequences as control. Again a GFP co-transfected plasmid was used, and the white arrows show the transfected neurons (scale bar 20 µm). The right panel shows quantification of PDCD4 levels, detecting a decrease of 0.66 fold compared to the control (p value (**) ≤0.01, two tailed Mann Whitney test, n=3 with 3 technical replicates, error bars: SEM). **(C)** Fluorescent image of DRG neurons were PDCD4 was knockdown using 4 cell permeable siRNA sequences against PDCD4 (Dharmacon Accell siRNA SMARTPOOL) or a mix of 4 Accell scrambled siRNA sequences as control. Tubulin and PDCD4 expression is shown (scale bar 20 µm). The right panel shows quantification of PDCD4 levels detecting a decrease of 0.65 fold compared to the control (p value (*) ≤0.02, two tailed Mann Whitney test, n=3 with 3 technical replicates, error bars: SEM). **(D)** To test if the siRNA probes or plasmids have an effect on total PDCD4 protein levels, we transfect N2A cell line, as a high transfection efficiency model. PDCD4 expression is shown by western blot as the upper bands, while lower bands correspond to actin as a loading control. The right panel shows relative quantification of PDCD4 Western Blots bands (p value (***) ≤0.001 and p value (*) ≤0.02, two tailed Mann Whitney test, n=3 with 2 technical replicates, error bars: SEM).

**Figure S2.** PDCD4 expression silenced by inducible shRNA in the PC12 cell line. Inducible PDCD4 silencing in PC12 cells was achieved by lentiviral transfection and confirmed by semi-quantitative RealTime PCR and immunofluorescence. **(A)** PDCD4 expression was followed at different time points of NGF-induced neuron differentiation in wt PC12 cells using immunofluorescence. **(B)** Neuron-like phenotype achieved in PC12 cells after 72 h of NGF exposure, evidenced by phase contrast microscopy. **(C)** PDCD4 expression evaluated by immunofluorescence was analyzed in PC12-shScrambled and PC12-shPDCD4 cells after NGF exposure alone or with shRNA expression induction by doxycycline (DOX). Illustrative images are shown. **(D)** PDCD4 signal quantification shows a high decrease in PDCD4 only in PC12-shPDCD4 exposed to NGF+DOX (p value (**) <0.01, One-way ANOVA with post-hoc Tukey test; n=2 with almost 150 cells analyzed per replicate). **(E)** PDCD4 silencing was also confirmed by semi-quantitative RealTime PCR (p value (***) <0.001, Student’s t test, n=3, error bars: SD). **(F)** GFP expression was also monitored by immunofluorescence as an indication of shRNA expression induction by DOX. Illustrative images are shown. **(G)** Quantification of GFP signal shows insignificant levels in absence of DOX while considering levels when is present. Differences were statistically significant (p value (**) <0.01, one-way ANOVA with post-hoc Tukey test, n=2 with almost 150 cells analyzed per replicate). In A-C and F scale bars means 20 µm.

**Figure S3.** RNA-Seq and Ribo-Seq data were analyzed individually by edgeR. **(A)** Ribosomal footprints periodicity was explored comparing 5’-end read mapping distribution among the three codon nucleotides. Each dot represents a fraction of reads in each position for each sample (transcriptome RNA-Seq derived and translatome Ribo-Seq derived). For translatome samples the first position in the codon tends to be enriched but for transcriptome samples distribution tends to be uniform. **(B)** Mapping distribution among mRNA features was studied comparing 5’UTR, CDS and 3’-UTR expression and comparing between transcriptomes and translatomes. In the first, the three regions are expressed while for translatome samples CDS is preferentially expressed over UTRs regions. **(C)** Inter-replicate Pearson correlation value is shown for transcriptome and translatome samples, in the two conditions (shScrambled and shPDCD4). **(D)** and **(E)** Volcano and MA plot, respectively, for transcriptome compartment (RNA-Seq). **(F)** and **(G)**, same as (D) and (E) but for translatome compartment (Ribo-Seq). In D-G red and green dots indicate differentially expressed genes, up- and down-regulated, respectively (|fold change| >2 and p value <0.05).

**Figure S4.** Heatmap of potential PDCD4 targets. RNA-Seq and Ribo-Seq expression levels in PDCD4 presence and absence (shScrambled and shPDCD4, respectively) is shown for the 267 putative PDCD4 mRNA targets.

**Figure S5.** Quantification and comparison of protein abundance in PDCD4 presence and absence (shScrambled and shPDCD4, respectively) by label-free quantitative proteomics. **(A)** Scatter plot comparing protein abundance (LFQ/iBAQ values) in shScrambled and shPDCD4 conditions. Red and green dots indicate differentially expressed proteins, up- and down-regulated respectively (p value <0.05 estimated by ANOVA). PDCD4 is indicated in the scatter where the knock-down could be also evidenced. **(B)** Correlation between fold change values estimated by proteomics and Ribo-Seq for 87 differentially expressed proteins at the proteome that also show the same direction of change in the Ribo-Seq data.

**Figure S6.** Functional protein association network by STRING for PDCD4 putative translational targets defined by translational efficiency criteria **(A)** and for the opposite direction genes **(B)**. In (A) the 4 functional-related clusters discussed in the text are indicated: Cluster 1 in blue, highlighting genes associated to mitosis and cell cycle; Cluster 2 in red, highlighting genes associated to the nucleus; Cluster 3 correspond to mitochondrial activity and Cluster 4 in yellow, highlighting genes associated to protein export. Disconnected nodes are hidden in (A) but shown in (B) for illustrative reasons.

**Figure S7.** Lentiviral shPDCD4 on cortical neurons cultured *in vitro* upregulates total levels of NFKB2 measured by Western Blot. **(A)** Cortical neurons were transfected with increasing amounts of lentiviral particles (+ and ++) containing shPDCD4 sequences, or with vehicle (no lentiviral particles). Western blot bands for PDCD4, NFKB2 and TUBB3 as non-target control are shown in each case. **(B)** Relative quantitation of PDCD4 and NFKB2 levels in each condition is shown. While levels of PDCD4 descend down to three-fold with increasing lentiviral particles, NFKB2 levels increase more than two fold.

**Figure S8.** Comparison of down-regulated mRNAs targets after PDCD4 knockdown with previously reported axonal transcriptomes. **(A)** Venn diagram showing the intersection between TE down-regulated mRNAs by PDCD4 and axonal transcriptomes described in Figure 4B. Separate and overlapping expressions between samples are shown. Only transcripts with a level of expression of TPM ≥ 1 were considered. **(B)** The table shows the type of neuron used in each study, the total genes detected and the number of common genes between potential PDCD4 targets (or potential PDCD4 targets related to axonal growth) and each axonal transcriptome. The EASE Score (a modified Fisher Exact *p-value*) is also shown, which indicates gene-enrichment.

**Supplementary Tables Legends.**

**Table S1.** Total number of reads sequenced and mapped in both transcriptome and translatome samples.

**Table S2.** Average RPKM expression values, log_2_ fold change in translational efficiency and p values for putative PDCD4 targets.

**SI References**

[Garcez PP, Guillemot F, Dajas-Bailador F. 2016. Study of miRNA Function in the Developing Axons of Mouse Cortical Neurons: Use of Compartmentalized Microfluidic Chambers and In Utero Electroporation. *MicroRNA Technologies* 59–71.](http://paperpile.com/b/bm7mcc/rJ5Y) <http://dx.doi.org/10.1007/7657_2016_12>[.](http://paperpile.com/b/bm7mcc/rJ5Y)

[Jensen LJ, Kuhn M, Stark M, Chaffron S, Creevey C, Muller J, Doerks T, Julien P, Roth A, Simonovic M, et al. 2009. STRING 8--a global view on proteins and their functional interactions in 630 organisms. *Nucleic Acids Res* **37**: D412–6.](http://paperpile.com/b/bm7mcc/Bx1R7)

[Langmead B, Salzberg SL. 2012. Fast gapped-read alignment with Bowtie 2. *Nat Methods* **9**: 357–359.](http://paperpile.com/b/bm7mcc/bpZ1R)

[Liao Y, Smyth GK, Shi W. 2014. featureCounts: an efficient general purpose program for assigning sequence reads to genomic features. *Bioinformatics* **30**: 923–930.](http://paperpile.com/b/bm7mcc/c4WcF)

[Liu X, Cheng Y, Yang J, Krall TJ, Huo Y, Zhang C. 2010. An essential role of PDCD4 in vascular smooth muscle cell apoptosis and proliferation: implications for vascular disease. *Am J Physiol Cell Physiol* **298**: C1481–8.](http://paperpile.com/b/bm7mcc/o2vu)

[Livak KJ, Schmittgen TD. 2001. Analysis of Relative Gene Expression Data Using Real-Time Quantitative PCR and the 2−ΔΔCT Method. *Methods* **25**: 402–408.](http://paperpile.com/b/bm7mcc/ycMr) <http://dx.doi.org/10.1006/meth.2001.1262>[.](http://paperpile.com/b/bm7mcc/ycMr)

[Lucci C, Mesquita-Ribeiro R, Rathbone A, Dajas-Bailador F. 2020. Spatiotemporal regulation of GSK3β levels by miRNA-26a controls axon development in cortical neurons. *Development* **147**.](http://paperpile.com/b/bm7mcc/ALjj) <http://dx.doi.org/10.1242/dev.180232>[.](http://paperpile.com/b/bm7mcc/ALjj)

[Parker SS, Mandell EK, Hapak SM, Maskaykina IY, Kusne Y, Kim J-Y, Moy JK, St John PA, Wilson JM, Gothard KM, et al. 2013. Competing molecular interactions of aPKC isoforms regulate neuronal polarity. *Proc Natl Acad Sci U S A* **110**: 14450–14455.](http://paperpile.com/b/bm7mcc/9Dho)

[Robinson MD, McCarthy DJ, Smyth GK. 2010. edgeR: a Bioconductor package for differential expression analysis of digital gene expression data. *Bioinformatics* **26**: 139–140.](http://paperpile.com/b/bm7mcc/ezyHC)

[Schindelin J, Arganda-Carreras I, Frise E, Kaynig V, Longair M, Pietzsch T, Preibisch S, Rueden C, Saalfeld S, Schmid B, et al. 2012. Fiji: an open-source platform for biological-image analysis. *Nat Methods* **9**: 676–682.](http://paperpile.com/b/bm7mcc/9xVRz)

[Xiao Z, Zou Q, Liu Y, Yang X. 2016. Genome-wide assessment of differential translations with ribosome profiling data. *Nat Commun* **7**: 11194.](http://paperpile.com/b/bm7mcc/q9v5z)

[Zhou L, Lim Q-E, Wan G, Too H-P. 2010. Normalization with genes encoding ribosomal proteins but not GAPDH provides an accurate quantification of gene expressions in neuronal differentiation of PC12 cells. *BMC Genomics* **11**: 75.](http://paperpile.com/b/bm7mcc/vzSr) <http://dx.doi.org/10.1186/1471-2164-11-75>[.](http://paperpile.com/b/bm7mcc/vzSr)
